# Description of T-Cell and Monocyte Populations in the Circulation of People with HIV Prior to AIDS-NHL Diagnosis

**DOI:** 10.3390/cells14201608

**Published:** 2025-10-16

**Authors:** Laura E. Martínez, Begoña Comin-Anduix, Miriam Güemes-Aragon, Javier Ibarrondo, Roger Detels, Matthew J. Mimiaga, Marta Epeldegui

**Affiliations:** 1 UCLA AIDS Institute, University of California, Los Angeles, CA 90095, USA; lauramartinez@mednet.ucla.edu (L.E.M.); fibarrondo@mednet.ucla.edu (J.I.); 2 Department of Obstetrics and Gynecology, David Geffen School of Medicine, University of California, Los Angeles, CA 90095, USA; 3 Jonsson Comprehensive Cancer Center, University of California, Los Angeles, CA 90095, USA; bcomin@g.ucla.edu (B.C.-A.); mguemes@mednet.ucla.edu (M.G.-A.); 4 Ahmanson Translational Theranostics Division, Department of Molecular and Medical Pharmacology, University of California, Los Angeles, CA 90095, USA; 5 Division of Surgical Oncology, Department of Surgery, University of California, Los Angeles, CA 90095, USA; 6 Department of Hematology and Oncology, David Geffen School of Medicine, University of California, Los Angeles, CA 90095, USA; 7 Department of Epidemiology, Fielding School of Public Health, University of California, Los Angeles, CA 90095, USA; detels@ucla.edu (R.D.); mmimiaga@ph.ucla.edu (M.J.M.)

**Keywords:** HIV, unsupervised clustering analysis, CD20^+^ T-cells, CD14^+^ monocytes, pre-lymphoma, AIDS-NHL

## Abstract

People with HIV (PWH) are at an increased risk for AIDS-associated non-Hodgkin lymphoma (AIDS-NHL); however, the immune signatures underlying this risk are not well understood. In this study, we utilized mass cytometry by time-of-flight (CyTOF) to analyze T-cells and monocytes in the PBMCs of treatment-naïve PWH, including those 3 to 36 months before an AIDS-NHL diagnosis (HIV-positive pre-NHL), as well as people without HIV (PWoH). Mass cytometry is an advanced single-cell analysis platform that combines flow cytometry principles with mass spectrometry. Unlike conventional flow cytometry, this technology employs antibodies conjugated to unique metal isotopes instead of fluorescent markers, enabling simultaneous measurement of over 40 distinct cellular markers per individual cell without spectral overlap limitations. Participants were enrolled at the Los Angeles site of the MACS/WIHS Combined Cohort Study (MWCCS). Unsupervised clustering and Uniform Manifold Approximation and Projection (UMAP) analysis identified CD3^+^ T-cell and CD14^+^ monocyte metaclusters, and Spearman’s rank correlation assessed their relationships with B-cell subsets exhibiting aberrant phenotypes. We observed elevated levels of CD8^+^CD20^+^ T-cells, CD8^+^CD14^+^ T-cells, and M2-like CD14^+^CD163^+^ monocytes in HIV-positive pre-NHL individuals compared to HIV-negative controls. Positive correlations were found between CD19^+^ AICDA^+^ cMYC^+^ B-cells and M1-like CD14^+^cMYC^+^ monocytes (metacluster, MC02), and between metaclusters of CD8^+^PD-1^+^CD27^+^CXCR4^−^ T-cells (MC05) and CD4^+^FoxP3^+^PD-1^+^CD27^+^CD28^+^CXCR4^−^ ICOS^+^ T-cells (MC08). In addition, a different CD19^+^ B-cell metacluster (FoxP3^+^AICDA^+^cMYC^+^) was positively associated with a metacluster of CD8^+^PD-1^+^CD27^+^CD28^+^CXCR4^+^ T-cells (MC03). Moreover, the metacluster of CD8^+^PD-1^+^CD27^+^CXCR4^−^ T-cells (MC05) negatively correlated with M2-like CD14^+^CD163^+^ monocytes (MC06), while CD8^+^CD14^+^ T-cells positively correlated with AICDA^+^ Bregs and IL-10^+^ B-regs in HIV-positive pre-NHL individuals. Unsupervised analysis revealed increased frequencies of CD8^+^CD20^+^ T-cells in HIV-positive individuals compared to HIV-negative controls. These immune alterations provide valuable insights into potential biomarkers for early detection, monitoring, and therapeutic strategies for AIDS-NHL.

## 1. Introduction

Chronic HIV infection significantly elevates the risk of B-cell non-Hodgkin lymphoma (NHL) [1]. While combination antiretroviral therapy (cART) has notably improved survival rates for people with HIV (PWH), NHL remains a leading cause of morbidity and mortality in this population, even after the widespread adoption of cART [1]. HIV-induced immune suppression promotes the unchecked proliferation of abnormal cells, increasing susceptibility to various types of lymphoma, including Burkitt lymphoma (BL), diffuse large B-cell lymphoma (DLBCL), primary central nervous system lymphoma (PCNSL), plasmablastic lymphoma (PBL), and primary effusion lymphoma (PEL) [1]. Lymphomas in PWH are driven by chronic antigen exposure, genetic mutations, dysregulated pro-inflammatory cytokine production, and loss of immune control during co-infection with oncogenic viruses like Epstein-Barr virus (EBV) and Kaposi’s sarcoma-associated herpesvirus (KSHV), both of which play a role in lymphoma development [2].

Germinal center (GC) dysregulation represents a fundamental mechanism underlying B-cell lymphomagenesis, serving as the critical microenvironmental niche where normal B-cell maturation processes become hijacked by malignant transformation [3,4,5]. In pathological states of PWH, chronic immune activation disrupts normal GC architecture, leading to aberrant follicular structures characterized by uncontrolled BCL-6 and c-MYC expression that drives the proliferation of cells and blocks terminal differentiation [6,7,8,9,10]. This dysregulated environment is impacted by follicular helper T-cell (Tfh) dysfunction, creating a cascade of immunological abnormalities: Elevated PD-1^high^ Tfh cell populations that fail to provide appropriate B-cell signals [11,12,13], impaired immune surveillance, and the establishment of immune-privileged niches through upregulation of checkpoint ligands (PD-L1, CD80/CD86) on antigen-presenting cells [14]. Collectively, these processes create optimal conditions for clonal evolution and malignant cell expansion.

Chronic immune activation and inflammation caused by HIV, along with immune responses in the pre-tumor microenvironment, may promote the early development of lymphoma [15,16,17]. HIV-induced decline in CD4^+^ T-cells weakens immune surveillance, increasing lymphoma risk, particularly in individuals not receiving cART [1]. However, even with cART, immune dysregulation persists, and low CD4^+^ T-cell counts in PWH remain associated with a heightened risk of lymphoma subtypes such as BL, DLBCL, PEL, and PCNSL, often linked to co-infection with EBV and KSHV [1]. While cART improves immune function by increasing CD4^+^ T-cells and reducing viral load, chronic immune activation persists, particularly in CD8^+^ T-cells, driving B-cell dysregulation and promoting lymphoma development [18,19,20].

Recent studies have highlighted a subset of CD8^+^ T-cells that express CD14 and may play an immunomodulatory role, particularly in response to chronic inflammation or persistent antigen exposure, such as during hepatic viral responses and tumor infiltration in cancer [21]. These cells can also be modulated by LPS, a marker of microbial translocation [21]. Additionally, emerging evidence suggests that CD20-expressing T-cells, which typically express CD20 on B-cells, are involved in immune dysregulation, potentially fostering an environment conducive to tumorigenesis [22,23,24,25,26,27]. CD20^+^ T-cells have been identified in lymphoid tissues of PWH, especially those with a history of chronic immune activation and inflammatory responses [28,29,30,31]. This underscores the need for further investigation into the roles of these specific T-cell populations in HIV pathogenesis and related malignancies.

Monocytes and macrophages play key roles in the inflammatory response and immune dysregulation in chronic HIV infection [32,33,34,35,36,37,38,39,40,41]. In HIV infection, persistent monocyte activation increases the production of TNF-α, IL-1β, IL-6, and soluble CD14 (sCD14), creating a chronic inflammatory environment that drives both B-cell dysregulation and immune suppression [37,38,40,42,43,44]. Monocyte-driven immune suppression and macrophage polarization contribute to viral reservoirs and the development and progression of AIDS-associated malignancies in PWH [36,37,40,43,44,45,46,47,48].

In a recent study, we performed Uniform Manifold Approximation and Projection (UMAP) and unsupervised clustering to analyze B-cells in HIV-positive pre-NHL samples [49]. We observed that certain B-cell populations were significantly elevated in HIV-positive cART-naïve samples compared to HIV-negative controls. These included a potentially clonal B-cell population (CD20^+^CXCR4^+^CXCR5^+^cMYC^+^AICDA^+^) and a germinal center-like B-cell cluster (CD19^−^CD20^+^CXCR4^+^Bcl-6^+^PD-L1^+^cMYC^+^) circulating in HIV-positive pre-NHL (cART-naïve) individuals [49]. In addition, significantly higher levels of CD19^+^CD24^hi^CD38^hi^ B regulatory cells with cMYC^+^ and AICDA^+^ phenotypes were identified in HIV-positive pre-NHL compared to HIV-positive cART-naïve individuals [49].

This study aimed to identify and characterize CD3^+^ T-cells and CD14^+^ monocytes in the circulation of PWH, focusing on HIV-positive cART-naïve individuals who went on to develop AIDS-NHL within 3 to 36 months prior to their diagnosis (pre-NHL). The results were compared with HIV-positive cART-naïve individuals who did not go on to develop AIDS-NHL and HIV-negative controls. Studying cART-naïve individuals is important because it allows us to better understand the underlying biology of HIV-associated lymphoma. A substantial proportion of AIDS-NHL cases are diagnosed around the same time as HIV infection, when patients are still cART-naïve. Since many individuals are unaware of their HIV status until after infection, critical immunologic and oncogenic processes may unfold in the cART-naïve state. Although cART improves immune cell recovery, the cells often do not return to baseline ‘normal’ levels, highlighting the continued importance of studying this group [50].

By analyzing T-cell and monocyte populations, we sought to uncover markers of immune dysfunction that may serve as early indicators of AIDS-NHL in this high-risk group. We hypothesized that the pre-NHL development stages in PWH involve coordinated dysfunction across multiple immune compartments, including T-cell exhaustion, B-cell dysregulation, innate immune activation, and immune checkpoint pathways that can be detected through integrated analysis of specific biomarker signatures and immunophenotyping.

Using UMAP and unsupervised clustering, we characterized CD3^+^ T-cell and CD14^+^ monocyte metaclusters, exploring their relationships with previously identified B-cell subsets expressing AICDA and cMYC [49]. Our findings reveal key immune subsets (CD8^+^CD20^+^ T-cells, CD8^+^CD14^+^ T-cells, and M2-like CD14^+^CD163^+^ monocytes) in HIV-positive pre-NHL individuals that could offer novel biomarkers for early detection and risk stratification of AIDS-NHL, advancing our understanding of the immune dysregulation driving lymphomagenesis in PWH. These insights pave the way for improved diagnostic tools and targeted therapeutic strategies aimed at mitigating lymphoma progression in this vulnerable population.

## 2. Materials and Methods

### 2.1. Ethics Approval Statement

The MWCCS was approved by the UCLA Institutional Review Board (IRB) to ensure the safety and protection of the participants involved in human subjects research (IRB# 20-002292); all participants provided written informed consent. All specimens and clinical data provided by the MWCCS were de-identified.

### 2.2. Study Participants

PBMCs were obtained from three groups: HIV-negative individuals (n = 10), HIV-positive and cART naïve individuals who did not develop NHL (n = 20), and HIV-positive individuals who developed NHL (n = 10) (HIV-positive pre-NHL, cART-naïve). PBMC samples from HIV-positive pre-NHL (cART-naïve) individuals were selected from visits prior to their AIDS-NHL diagnosis with a median of 12 months (range of 6 to 36 months) and were matched by HIV status, age, and recruitment period [49]. The 6- to 36-month sampling window offered a biologically relevant timeframe for identifying predictive markers of B-cell lymphoma development, which is supported by our prior studies evaluating cytokines and B-cell markers as predictors of AIDS-NHL [51,52,53].

Among the information provided for HIV-positive pre-NHL cases (n = 10), seven cases subsequently developed NHL, with extranodal presentation in five cases and nodal presentation in two cases. All cases were of B-cell lymphoma origin. Given the limited sample size of pre-NHL cases, our analysis focused on metacluster analysis and phenotype characterization of B-cell [49], T-cell, and monocyte populations identified through unsupervised clustering across grouped data from all pre-NHL cases. Individual case-by-case immunophenotypic comparisons were not performed and represent an important objective for future studies with larger cohorts.

All samples were sourced from viable cryopreserved PBMC vials stored at the UCLA site of the Multicenter AIDS Cohort Study (MACS). MACS, a long-term prospective study of HIV and AIDS that began in 1984, includes adult gay and bisexual men from four MACS sites in U.S. metropolitan areas: Baltimore/Washington (Johns Hopkins University), Chicago (Northwestern University), Los Angeles (UCLA), and Pittsburgh (University of Pittsburgh). In 2019, MACS merged with the Women’s Interagency HIV Study (WIHS) to form the MACS/WIHS Combined Cohort Study (MWCCS). The PBMC samples were specifically from MACS visits conducted between 1985 and 2002. The MACS includes over 200 AIDS-related lymphoma cases and appropriate HIV-negative and HIV-positive controls [54].

### 2.3. Mass Cytometry Antibody Panel

The mass cytometry panel was designed using Maxpar Panel Designer software (v2.0.1) (Fluidigm/Standard BioTools, South San Francisco, CA, USA), accounting for metal oxidation, antibody signaling, and antibody tolerance. The CyTOF panel included markers to immunophenotype B-cells and their activation states: CD19, CD20, CD24, CD38, CD40, CD71, and HLA-DR. It also featured lineage markers for T-cells (CD3, CD4, CD8) and monocytes (CD14, CD11b, CD163) [49]. The panel included chemokine receptors (CXCR4, CXCR3, CXCR5, CCR5), B-cell antibody secretion molecules (IgG, IgM, Ig kappa and lambda light chain), and checkpoint/signaling molecules (PD-1, PD-L1, PD-L2, ICOS, CD80, CD86, CD27, CD28, CD40L, CTLA-4) [49]. The panel also included transcription factors (Bcl-6, FoxP3), the immunoregulatory cytokine IL-10, oncogenic markers (CD10, cMYC, AICDA), the EBV marker latent membrane protein-1 (LMP1), and a marker of active HIV infection (KC57) [49]. Antibodies were either purchased pre-conjugated from Fluidigm/Standard BioTools (South San Francisco, CA, USA) or conjugated at the UCLA Jonsson Comprehensive Cancer Center (JCCC) and the Center for AIDS Research Flow Cytometry Core Facility (Los Angeles, CA, USA). Metal-isotope-labeled antibodies were conjugated using the MaxPar X8 Multimetal Labeling Kit (Standard BioTools Inc., South San Francisco, CA, USA) for lanthanides and the MaxPar MCP9 Antibody Labeling Kit (Standard BioTools Inc., South San Francisco, CA, USA) for cadmium metal isotopes, following the manufacturer’s protocol (Fluidigm/Standard BioTools). Each conjugated antibody was quality-checked and titrated for optimal staining using PBMCs from healthy blood donors (UCLA Virology Core, Los Angeles, CA, USA) and/or B-cell lymphoma cell lines (Ramos, Raji, and 2F7). The extracellular and intracellular antibody panel details are summarized in Supplementary Table S1.

### 2.4. Mass Cytometry Staining

Cryopreserved PBMCs were thawed into complete RPMI-1640 medium containing 10% FBS supplemented with 1% penicillin and streptomycin (Gibco, Life Technologies Corp., Grand Island, NY, USA) and recovered for 5 min at room temperature. PBMCs were then centrifuged for 5 min at 1500 rpm at room temperature. Cell pellets were resuspended in 1 mL of complete RPMI, treated with 100 µL of DNase I (2.27 KU/mL; Catalog No. D5025-150KU; Sigma-Aldrich, St. Louis, Missouri) and incubated for 30 min at 37 °C in a 5% CO_2_ incubator. After the 30-min incubation, 9 mL of complete RPMI was added for a total of 10 mL; cells were resuspended, and an aliquot was taken to count cells. Cells were centrifuged for 5 min at 1500 rpm at room temperature to wash out DNAse I. Cell pellets were then resuspended in 1 mL of 5 µM Cell-ID Cisplatin (Catalog No. 201064; Standard BioTools Inc., South San Francisco, CA, USA). The cisplatin was then quenched by adding Maxpar Cell Staining Buffer (MCSB) (Fluidigm/Standard BioTools, Catalog No. 201068, Standard BioTools Inc., South San Francisco, CA, USA) at 5× volume. Fc block (10% BSA in 1× PBS) was filtered-sterilized and added to cells for a 10-min incubation at room temperature. For cell staining, we followed manufacturer-recommended protocols and best practices from Fluidigm (Standard BioTools, South San Francisco, CA, USA). Each cell sample was stained separately and individually (not pooled within cohort groups) using metal-conjugated antibodies for both extracellular and intracellular markers. The extracellular surface-staining antibody cocktail (Supplementary Table S1) was added in MCSB and incubated at room temperature for 30 min. For intracellular staining, cells were resuspended in FoxP3 fixation/permeabilization solution (Catalog No. 00-5523-00, ThermoFisher Scientific, Waltham, MA, USA) for 30 min at room temperature, washed with FoxP3 permeabilization buffer, and centrifuged at 800× *g* for 5 min at room temperature. The intracellular antibody cocktail (Supplementary Table S1) was added and incubated for 1 h at room temperature. After washing, cells were resuspended in 1 mL of 200 nM iridium intercalation solution (Ir191/193) (Cell-ID Intercalator-Ir-500 μM, Catalog No. 201192B, Standard BioTools Inc., South San Francisco, CA, USA) in MaxPar fix and perm buffer (Catalog No. 201067, Standard BioTools Inc., South San Francisco, CA, USA) and incubated overnight at 4 °C. The next day, cells were washed once in 1 mL of MCSB and twice in 1 mL of MilliQ ddH_2_O. Immediately before acquisition, samples were filtered through a 35 μm nylon mesh cell strainer in MilliQ ddH_2_O. Bead-based normalization using EQ four element calibration beads was applied immediately before acquisition to correct for signal drift in the generated FCS files. Cells were acquired at a rate of 400–600 events/second using a Helios Mass Cytometer (Fluidigm/Standard BioTools, South San Francisco, CA, USA) at the UCLA Jonsson Comprehensive Cancer Center (JCCC) and the Center for AIDS Research Flow Cytometry Core Facility. Batch correction was performed after sample acquisition using CyTOF software packages (v7.1) and tools (Fluidigm/Standard BioTools, South San Francisco, CA, USA).

### 2.5. Mass Cytometry Data Analysis

During data acquisition, some samples became clogged in the Helios Mass Cytometer and required re-running. Instead of concatenating these samples at the beginning of the acquisition, they were concatenated later in the data analysis pipeline using the OMIQ platform (www.omiq.ai; Dotmatics, Boston, MA, USA). The analysis included 10 HIV-negative PBMC samples (12 FCS files), 20 HIV-positive cART-naïve PBMC samples (22 FCS files), and 10 HIV-positive pre-NHL PBMC samples (11 FCS files), all of which were included in the final dataset.

FCS files were uploaded to OMIQ for analysis. Calibration beads were removed, and time stabilization (191Ir+ vs. time) was checked for anomalies. Singlets (193Ir vs. 191Ir) and viable single-cell events (194Pt+) were identified, and manual gating was applied to select CD3^+^ T-cells or CD14^+^ monocytes (see Supplementary Figure S1).

### 2.6. Dimensionality Reduction Using UMAP

For immune cell population analysis, total viable single cells were analyzed using Uniform Manifold Approximation and Projection (UMAP) and FlowSOM clustering algorithms with all panel markers (see Supplementary Table S1). We optimized the analysis by testing different neighbor values (i.e., neighbors = 8, 15, or 20) and FlowSOM cluster dimensions (x-dim and y-dim = 10 or 20) to find the optimal settings for metacluster separation and refinement. After determining the best UMAP and FlowSOM parameters, we performed unsupervised clustering multiple times (3×) with different random seeds each time to ensure consistent population identification before refining metacluster numbers, gating, and conducting statistical comparisons.

### 2.7. Unsupervised Clustering of CD3^+^ T-Cells

For unsupervised clustering of CD3^+^ T-cells, we subsampled to 75,000 cells per group (three groups: HIV-negative, HIV-positive cART-naïve, and HIV-positive pre-NHL (cART-naïve)) and used UMAP and FlowSOM. UMAPs were generated in OMIQ with 22 nearest neighbors, a minimum distance of 0.01, 2 components, Euclidean metric, and 200 epochs. FlowSOM clustering was performed with x-dim = 18, y-dim = 18, rlen = 10, and Euclidean distance.

### 2.8. Unsupervised Clustering of CD14^+^ Monocytes

For unsupervised clustering of CD14^+^ monocytes, we followed a similar workflow: equal subsampling to 75,000 cells per group (HIV-negative, HIV-positive cART-naïve, and HIV-positive pre-NHL (cART-naïve)) and applying UMAP and FlowSOM. UMAPs were created in OMIQ with 22 nearest neighbors, a minimum distance of 0.01, 2 components, Euclidean metric, and 200 epochs. FlowSOM clustering used x-dim = 18, y-dim = 18, rlen = 10, and Euclidean distance.

### 2.9. Statistical Analysis

Cell population frequencies were determined using either manual gating or FlowSOM clustering algorithms. Differentially abundant CD3^+^ T-cell or CD14^+^ monocyte populations were analyzed using a Mann–Whitney test (two-tailed, unpaired, and non-parametric) and/or the edgeR statistical program in OMIQ. Data analysis and visualization were conducted with OMIQ and GraphPad Prism 10.3.0. For heatmaps, median marker expression values between groups were compared using a Mann–Whitney test (two-tailed, unpaired, and non-parametric). We used two-sample or multiple comparison adjustments, including Benjamini-Hochberg correction. Statistical significance was set at *p* < 0.05 (* *p* < 0.05; ** *p* < 0.005; *** *p* < 0.0005), with “ns” indicating not significant.

## 3. Results

### 3.1. MWCCS Study Participants

Study participants were men, primarily white, non-Hispanic, as previously described in Martinez et al., 2024 [49]. All HIV-positive (n = 20) and HIV-positive pre-NHL (n = 10) samples were collected from individuals who were cART-naïve at the time of sample collection.

### 3.2. Characterization of CD3^+^ T-Cells by Unsupervised Clustering Analysis

PBMCs from 10 HIV-negative, 20 HIV-positive cART-naïve, and 10 HIV-positive pre-NHL participants were labeled with metal-tagged antibodies for mass cytometry by time-of-flight (CyTOF), as previously described [49]. The panel included lineage cell markers for CD19^+^ B-cells, CD3^+^ T-cells (CD4^+^ and CD8^+^ T-cells), and CD14^+^ monocytes; markers of B-cell activation and/or differentiation; and oncogenic markers (Supplementary Table S1). Before analysis, samples were normalized, and sample acquisition stability was confirmed (191Ir+ vs. time). Cells were gated using 191Ir+ vs. 193Ir+, doublets were discarded, and viable cells were gated as 194Pt- (Supplementary Figure S1A). UMAP plots of singlets and viable cells showed clear resolution of CD3^+^ T-cells (Supplementary Figure S1B), from which CD4^+^CD8^−^ T-cells, CD4^−^CD8^+^ T-cells; CD4^+^CD8^+^ T-cells, and CD3^+^ double-negative T-cells were identified (Supplementary Figure S1C). CD4^+^ T-cells were significantly lower in HIV-positive individuals, as expected, while CD8^+^ T-cells were significantly elevated in both HIV-positive cART-naïve and HIV-positive pre-NHL (cART-naïve) samples compared to HIV-negative samples (Supplementary Figure S1D). Supplementary Figure S2 shows histogram overlays of different markers of activation and inhibition analyzed in this study.

**Figure 1:**
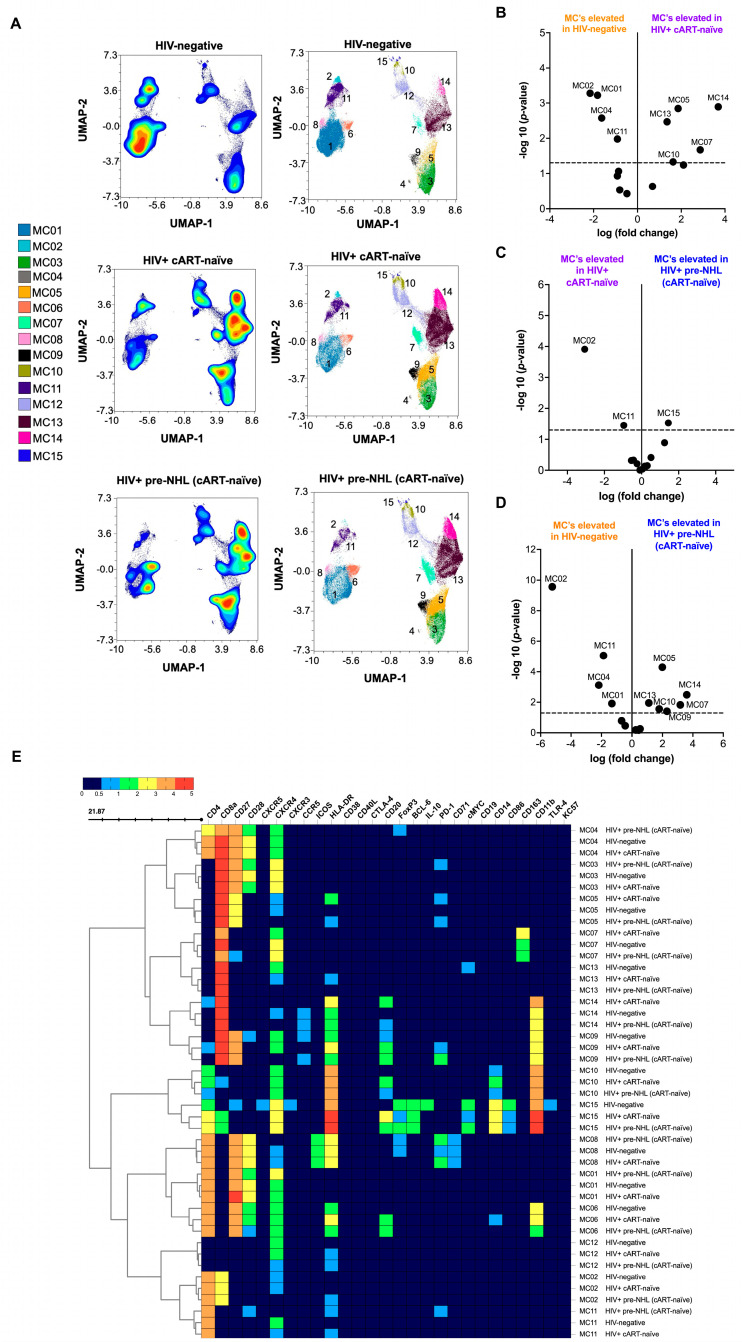
CD4^+^CD20^+^ T-cells and CD8^+^CD20^+^ T-cells are elevated in HIV-positive cART-naïve and HIV-positive pre-NHL (cART-naïve) compared to HIV-negative individuals. (**A**) Unsupervised clustering of CD3^+^ T-cells identified 15 metaclusters (MC01 to MC15) in samples from HIV-negative (n = 10), HIV-positive cART-naïve (n = 20), and HIV-positive pre-NHL (cART-naïve) (n = 10) groups. Contour plots of UMAPs were generated after subsampling 75,000 CD3^+^ T-cells (CD19^−^) from each group. Each metacluster is represented by a different color in the UMAP scatter plot (right). Volcano plots (**B**–**D**) show log fold-change (log FC) and adjusted *p*-values (*p* < 0.05) for significant differences in T-cell metaclusters across groups. Significant metaclusters are highlighted and shown above the −log10 (*p*-value) 0.05 threshold (dotted line) for comparisons between HIV-negative and HIV-positive cART-naïve samples (**B**) and between HIV-positive cART-naïve and HIV-positive pre-NHL (cART-naïve) samples (**C**). For comparison between HIV-negative and HIV-positive pre-NHL (cART-naïve) samples, significant metaclusters are shown in (**D**). (**E**) Heatmap showing median marker expression values for all identified metaclusters in (**A**), summarizing concatenated data of HIV-negative, HIV-positive cART-naive, and HIV-positive pre-NHL (cART-naïve) samples.

We then performed unsupervised clustering analysis of CD3^+^ T-cells to describe phenotypically distinct T-cell populations in PWH and the period preceding the appearance of lymphoma (HIV-positive pre-NHL) (6 to 36 months, with a median of 12 months) [49]. For in-depth and high-dimensional analysis, we performed unsupervised clustering of 75,000 CD3^+^ T-cells (CD3^+^CD19^−^) from concatenated FCS data files for each group after mass cytometry: 10 HIV-negative (12 data files), 20 HIV-positive cART-naïve (22 data files), and 10 HIV-positive pre-NHL (cART-naïve) samples (11 data files) (Figure 1). From there, uniform manifold approximation and projection (UMAP) for dimension reduction and FlowSOM clustering algorithms were conducted in OMIQ (Supplementary Figure S3). High-dimensional analysis of CD3^+^ T-cells identified 15 metaclusters in HIV-negative, HIV-positive cART-naïve, and HIV-positive pre-NHL samples (Figure 1A and Supplementary Figure S4).

Significant differences in T-cell metaclusters were observed between the three groups. Volcano plots were generated comparing HIV-negative to HIV-positive cART-naïve samples, HIV-positive cART-naïve to HIV-positive pre-NHL, and HIV-negative to HIV-positive pre-NHL (Figure 1B–D). The significant differences in T-cell populations were further supported by adjusted *p*-values (*p* < 0.05) and log fold-change (log FC) values indicated in the volcano plots. Overall, five metaclusters were significantly elevated in HIV-positive cART-naïve compared to HIV-negative (above the threshold of −log10 (*p*-value < 0.05)) (Figure 1B). One metacluster was significantly elevated in HIV-positive pre-NHL compared to HIV-positive cART-naïve (Figure 1C), and six metaclusters were elevated in HIV-positive pre-NHL compared to HIV-negative samples (Figure 1D). Figure 1E summarizes the marker expression values of all identified metaclusters across groups, highlighting key differences in T-cell metaclusters between HIV-negative, HIV-positive cART-naïve, and HIV-positive pre-NHL samples, and each phenotype is described in Supplementary Table S2.

### 3.3. CD8^+^CD20^+^ T-Cells Are Significantly Elevated in HIV-Positive cART-Naïve Compared to HIV-Negative Individuals

A group of metaclusters was significantly elevated in the HIV-positive groups, particularly CD8^+^CD20^+^ T-cells (Figure 1E). The results showed a significant elevation in these metaclusters in both HIV-positive cART-naïve and HIV-positive pre-NHL (cART-naïve) samples compared to HIV-negative samples. In metaclusters elevated in HIV-positive cART-naïve compared to HIV-negative, we found that they were comprised of CD4^+^CD8^+^CD20^+^CXCR4^−^CCR5^−^CD11b^+^HLA-DR^+^ (MC14), CD8^+^CD27^+^ CXCR4^+^CCR5^+^PD-1^+^HLA-DR^+^ (MC05), CD8^+^CXCR4^+^HLA-DR^+^ (MC13), a potential complex of CD8^+^ T-cells with CD163^+^ monocytes (MC07), and CD4^+^CD8^+^CD20^+^CXCR4^+^CD14^+^CD11b^+^HLA-DR^+^ (MC10) T-cells (Figure 1E and Supplementary Table S2).

The metacluster significantly elevated in HIV-positive pre-NHL compared to HIV-positive cART-naïve was a potential complex of T-cells and CD14^+^ monocytes (MC15) (Supplementary Table S2). Metaclusters elevated in HIV-positive pre-NHL compared to HIV-negative had the following phenotypes: CD8^+^CD27^+^CXCR4^−^PD-1^+^HLA-DR^+^ (MC05), CD8^+^CD20^+^CXCR4^−^CCR5^+^CD11b^+^HLA-DR^+^ (MC14), CD8^+^CXCR4^−^ (MC13), a potential complex of T-cells with CD14^+^ monocytes (MC10), a potential complex of T-cells with CD163^+^ monocytes (MC07), and CD8^+^CD20^+^CD27^+^CD28^−^CXCR4^−^CCR5^+^PD-1^+^CD11b^+^HLA-DR^+^ T-cells (MC09) (Supplementary Table S2).

### 3.4. CD8^+^CD14^+^ T-Cells Are Elevated in HIV-Positive Pre-NHL Individuals

**Figure 2:**
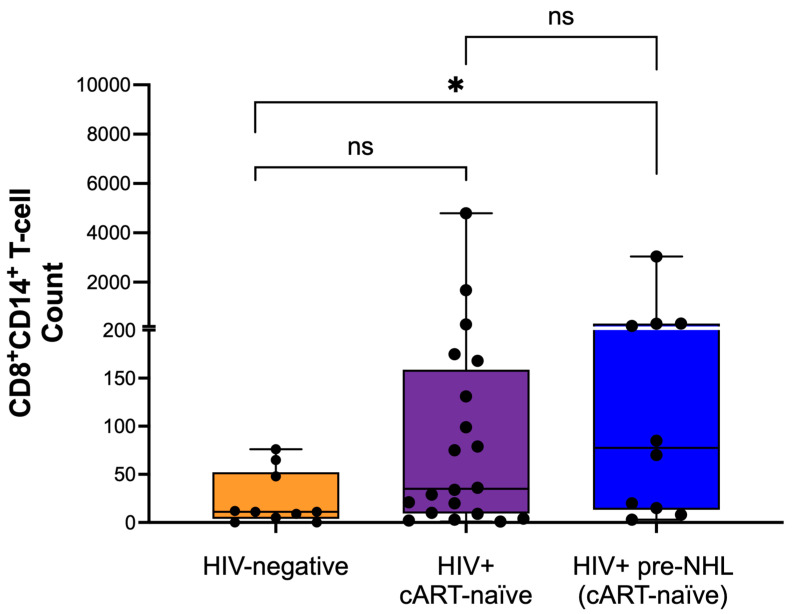
CD8^+^CD14^+^ T-cells are elevated in HIV-positive pre-NHL compared to HIV-negative individuals. CD8^+^CD14^+^ T-cell counts were determined for HIV-negative (n = 10), HIV-positive cART-naïve (n = 20), and HIV-positive pre-NHL (cART-naïve) (n = 10). Differences between each group were analyzed using a two-sided non-parametric test, Mann–Whitney U-test, with * *p* < 0.05 and ns, not significant.

Here, we sought to explore the role of CD8^+^CD14^+^ T-cells in HIV-positive cART-naïve, HIV-positive pre-NHL, and HIV-negative individuals. After manually gating CD8^+^CD14^+^ T-cells (Supplementary Figure S5), we compared frequencies across the different groups. Our results revealed that the numbers of CD8^+^CD14^+^ T-cells were significantly elevated in HIV-positive pre-NHL individuals compared to HIV-negative (Figure 2). No significant differences were observed between HIV-positive cART-naïve and HIV-positive pre-NHL. While there was an apparent difference in the numbers and spread of CD8^+^CD14^+^ T-cells across HIV-positive cART-naïve samples compared to HIV-negative samples, it did not reach statistical significance (Figure 2).

### 3.5. Characterization of CD14^+^ Monocytes by Unsupervised Clustering Analysis

**Figure 3:**
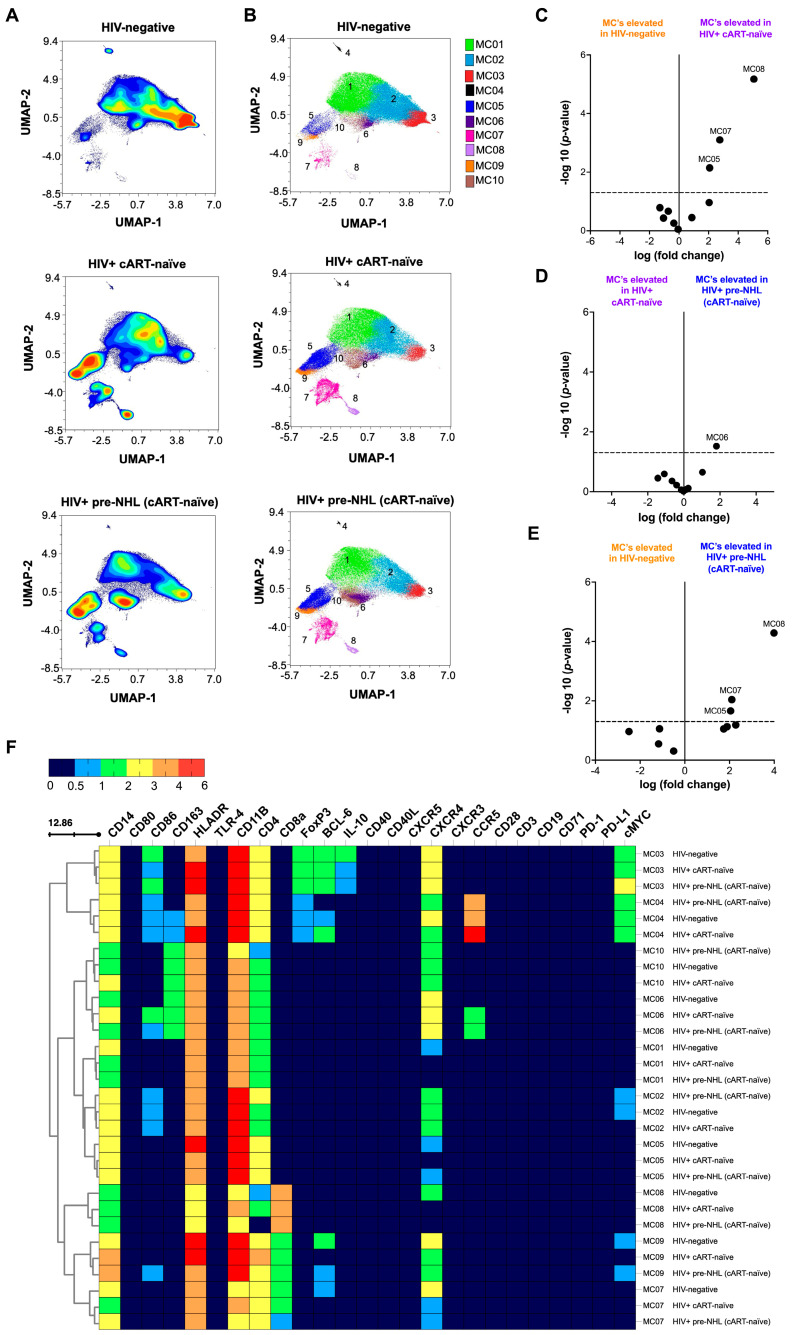
M2-like CD14^+^CD163^+^ monocytes are elevated in HIV-positive pre-NHL (cART-naïve) compared to HIV-positive cART-naïve individuals. (**A**) Unsupervised clustering of CD14^+^ monocytes identified nine metaclusters (MC01 to MC09) in samples from HIV-negative (n = 10), HIV-positive cART-naïve (n = 20), and HIV-positive pre-NHL (cART-naïve) (n = 10) groups. Contour plots of UMAPs were generated after subsampling 75,000 CD14^+^ monocytes (CD3^−^CD19^−^) from each group. (**B**) Each metacluster is represented by a different color in the UMAP plot. Volcano plots (**C**–**E**) show log fold-change (log FC) and adjusted *p*-values (*p* < 0.05) for significant differences in CD14^+^ monocyte metaclusters across groups. Three significant metaclusters are highlighted and shown above the −log10 (*p*-value) 0.05 threshold (dotted line) for comparisons between HIV-negative and HIV-positive cART-naïve samples (**C**). One significant metacluster is shown above the −log10 (*p*-value) 0.05 threshold (dotted line) for comparison between HIV-positive cART-naïve and HIV-positive pre-NHL (cART-naïve) samples (**D**). For comparison between HIV-negative and HIV-positive pre-NHL (cART-naïve) samples, three significant metaclusters are shown in (**E**). (**F**) Heatmap showing median marker expression values for all identified metaclusters in (**A**), summarizing concatenated data of HIV-negative, HIV-positive cART-naive, and HIV-positive pre-NHL (cART-naïve) samples.

To focus on monocytes, we excluded T-cells and B-cells (Supplementary Figure S6). Histogram plots of cell markers in CD14^+^ monocytes (CD14^+^CD3^−^CD19^−^) are summarized in Supplementary Figure S7. Unsupervised clustering of CD14^+^ monocytes from HIV-negative (n = 10), HIV-positive cART-naïve (n = 20), and HIV-positive pre-NHL (cART-naïve) (n = 10) groups identified nine distinct metaclusters (MC01 to MC09) (Figure 3A). UMAP plots showed clear differences in CD14^+^ monocyte subsets between the groups (Figure 3B and Supplementary Figure S8). Volcano plots showed significant differences in CD14^+^ monocyte metaclusters, with three metaclusters elevated in HIV-positive cART-naïve compared to HIV-negative samples (Figure 3C), one metacluster elevated in HIV-positive pre-NHL compared to HIV-positive cART-naïve samples (Figure 3D), and three metaclusters elevated in HIV-positive pre-NHL compared to HIV-negative samples (Figure 3E). These differences were statistically significant, with adjusted *p*-values (*p* < 0.05) and log fold-change (log FC) values above the −log10 (*p*-value) 0.05 threshold.

### 3.6. M2-like CD14^+^CD163^+^ Monocytes Are Elevated in HIV-Positive Pre-NHL Individuals

A heatmap summarizing the median marker expression for each metacluster highlights distinct expression profiles across groups is shown in Figure 3F and Supplementary Table S3. The three metaclusters elevated in HIV-positive cART-naïve compared to HIV-negative samples had the following phenotypes: CD14^+^HLA-DR^+^CD11b^+^CD4^+^CXCR4^−^ (MC08), CD14^+^HLA-DR^+^CD11b^+^CD4^+^BLC-6^−^CXCR4^+^ (MC07), and CD14^+^HLA-DR^+^CD11b^+^CD4^+^CXCR4^−^ (MC05) (Supplementary Table S3).

The metacluster elevated in HIV-positive pre-NHL compared to HIV-positive cART-naïve was CD14^+^CD86^+^CD163^+^HLA-DR^+^CD11b^+^CD4^+^CXCR4^+^CCR5^+^ (MC06) (Supplementary Table S3). Similarly, the three metaclusters elevated in HIV-positive pre-NHL compared to HIV-negative were the same metaclusters elevated in HIV-positive cART-naïve compared to HIV-negative samples (MC08, MC07, and MC05). These metaclusters elevated in HIV-positive pre-NHL had the following phenotypes: CD14^+^HLA-DR^+^CD11b^+^CD4^−^CXCR4^−^ (MC08), CD14^+^HLA-DR^+^CD11b^+^CD4^+^BCL-6^−^CXCR4^+^ (MC07), and CD14^+^HLA-DR^+^CD11b^+^CD4^+^CXCR4^+^ (MC05) monocytes. Monocytes in HIV-positive cART-naïve samples of MC05 had a loss of CXCR4 expression compared to HIV-positive pre-NHL and HIV-negative samples (Supplementary Table S3). Both CD14^+^ monocyte metaclusters of MC07 in HIV-positive cART-naïve and HIV-positive pre-NHL individuals did not express BCL-6 compared to HIV-negative individuals (Supplementary Table S3). Lastly, both CD14^+^ monocyte metaclusters of MC08 in HIV-positive cART-naïve and HIV-positive pre-NHL individuals did not express CXCR4 compared to HIV-negative individuals (Supplementary Table S3).

### 3.7. Correlations for CD3^+^ T-Cells, CD14^+^ Monocytes, and CD19^+^ B-Cell Metaclusters Identified by Unsupervised Clustering Analysis of PBMCs

HIV infection leads to chronic immune activation and dysregulation, affecting T-cells and monocytes, which are central to HIV-related diseases like lymphoma. To explore the role of immune dysregulation in HIV-positive cART-naïve and HIV-positive pre-NHL, we analyzed associations between T-cells, monocytes, and previously identified malignant B-cell phenotypes [49]. Understanding how this chronic inflammation contributes to lymphoma risk, especially in pre-NHL stages, can inform strategies to mitigate inflammation and reduce lymphoma risk. We first conducted correlation analyses between T-cells and B-cells using statistical comparisons (95% confidence intervals, Spearman correlation coefficients, and *p*-values (*p* < 0.05)), summarized in Supplementary Table S4.

### 3.8. Significant Immune Correlations Identified in HIV-Positive Pre-NHL Individuals

**Figure 4:**
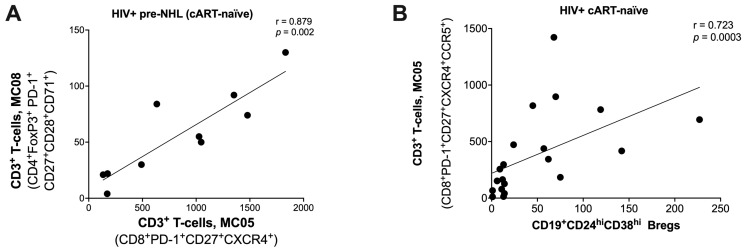
Significant Spearman’s rank correlations for significantly elevated metaclusters in HIV-positive pre-NHL (cART-naïve) and HIV-positive cART-naïve individuals. Spearman’s rank correlation plot for HIV-positive pre-NHL (cART-naïve): (**A**) CD3^+^ T-cells, MC08 (CD4^+^FoxP3^+^PD-1^+^CD27^+^CD28^+^CD71^+^ T-cells) vs. CD3^+^ T-cells, MC05 (CD8^+^PD-1^+^CD27^+^CXCR4^+^ T-cells). (**B**) Spearman’s rank correlation plot for HIV-positive cART-naïve: CD3^+^ T-cells, MC05 (CD8^+^PD-1^+^CD27^+^CXCR4^+^CCR5^+^ T-cells) vs. CD19^+^CD24^hi^CD38^hi^ Bregs. Differences between each group were analyzed using a two-sided non-parametric test, Mann–Whitney U-test. Spearman’s rank correlations are presented in Supplementary Table S4.

In the HIV-positive pre-NHL group, we identified significant immune correlations between T-cells and CD14^+^ monocytes (identified here by unsupervised clustering) and B-cell populations previously characterized in Martinez et al. 2024 [49]. CD19^+^ B-cells expressing AICDA and cMYC (metacluster 14, MC14) significantly correlated with M1-like CD14^+^CD86^+^ monocytes expressing cMYC (MC2). CD19^+^ B-cells expressing FoxP3, AICDA, and cMYC (MC21) correlated with CD8^+^PD-1^+^ T-cells (MC03) (shown in Supplementary Table S4). Moreover, CD8^+^PD-1^+^ T-cells (MC05) correlated positively with CD4^+^FoxP3^+^PD-1^+^ T-cells expressing CD27, CD28, ICOS, and CD71 (MC08) (Figure 4A) and negatively with M2-like CD14^+^CD86^+^CD163^+^ monocytes expressing CXCR4 and CCR5 (MC06) (Supplementary Table S4).

The analysis of CD8^+^CD14^+^ T-cells in HIV-positive pre-NHL revealed significant positive correlations with AICDA^+^ Bregs and IL-10^+^ B-regs (Supplementary Table S4). In HIV-positive cART-naïve individuals, B-regs significantly correlated with CD8^+^PD-1^+^ T-cells expressing CD27, CXCR4, and CCR5 (MC05) (Figure 4B and Supplementary Table S4).

## 4. Discussion

This study provides insights into the immune cell populations that are altered in HIV infection and pre-NHL. We observed distinct T-cell and monocyte subsets across the HIV-negative, HIV-positive cART-naïve, and HIV-positive pre-NHL (cART-naïve) groups, with significant elevation in specific immune populations in the HIV-positive groups.

### 4.1. Expansion of CD8^+^CD20^+^ T-Cells in HIV-Positive cART-Naïve and HIV-Positive Pre-NHL Individuals

HIV infection induces chronic immune activation, which can lead to the expansion of dysfunctional immune cell subsets. By unsupervised clustering analysis, we found significantly elevated metaclusters of CD8^+^CD20^+^ T-cells in both HIV-positive cART-naïve and HIV-positive pre-NHL (cART-naïve) individuals compared to HIV-negative controls. CD8^+^CD20^+^ T-cells have been identified in peripheral blood of healthy individuals [25] and in the progression of multiple sclerosis [27,55]. In the context of HIV, CD20^+^ T-cells have been found in both the bone marrow [56] and peripheral blood of PWH [29]. Förster et al. reported no significant difference in CD4^+^CD20^+^ T-cells as a percentage of lymphocytes between HAART-treated, untreated HIV patients, and healthy controls, but they did observe a relative increase in CD8^+^CD20^+^ T-cells in untreated HIV patients compared to both HAART-treated patients and healthy controls [29]. When Serra-Peinado et al. expressed CD4^+^CD20^+^ T-cells as a percentage of CD4^+^ T-cells, they found that untreated HIV patients had a higher proportion of CD4^+^CD20^+^ T-cells compared to HAART-treated patients and healthy controls [31]. While the literature on CD20^+^ T-cells remains limited, these findings highlight the need to further investigate their role in HIV pathogenesis.

In our cohort, CD8^+^CD20^+^ T-cells were consistently elevated in HIV-positive individuals compared to HIV-negative controls, highlighting a potential HIV-associated expansion of this population. While levels appeared modestly higher in HIV-positive pre-NHL compared to HIV-positive individuals without NHL, these differences did not reach statistical significance in metaclusters identified through unsupervised clustering analysis and should be interpreted cautiously given the small sample sizes (10 HIV-negative, 10 PWH pre-NHL, and 20 PWH). These findings suggest that CD8^+^CD20^+^ T-cell expansion is a feature of HIV infection, but larger studies are required to determine its relevance to NHL risk.

### 4.2. CD8^+^CD14^+^ T-Cells Are Elevated in HIV-Positive Pre-NHL Individuals

This study found elevated numbers of CD8^+^CD14^+^ T-cells in HIV-positive pre-NHL individuals compared to HIV-negative controls. This is a particularly interesting finding considering that CD8^+^CD14^+^ T-cells have recently been shown to be modulated by LPS [21]. In HIV infection, microbial translocation, the movement of gut-derived microbial substances like LPS into the bloodstream, is common in PWH with chronic inflammation and immune dysregulation [57]. Thus, these findings suggest that the increased presence of CD8^+^CD14^+^ T-cells in HIV-positive pre-NHL may be linked to ongoing immune activation and microbial translocation, contributing to chronic inflammation. In accordance with this, we have shown that markers of microbial translocation are elevated in pre-NHL [53] and that these markers can serve as prognostic indicators of AIDS-NHL [58].

### 4.3. Metaclusters of M2-like CD14^+^CD163^+^ Monocytes Are Elevated in HIV-Positive Pre-NHL Individuals

Our study highlights significant differences in immune cell subsets between HIV-positive pre-NHL and HIV-positive cART-naïve individuals. We observed elevated levels of M2-like CD14^+^CD163^+^ monocytes in HIV-positive pre-NHL, suggesting they may play a role in NHL pathogenesis. M2 monocyte phenotypes have been linked to inflammation, tissue remodeling, and immunosuppression [37,38], and may contribute to a microenvironment that promotes B-cell dysregulation. Moreover, the increased presence of CD14^+^CD163^+^ monocytes in HIV-positive pre-NHL aligns with previous studies showing altered immune profiles in individuals at higher risk for HIV-related malignancy [36,47,59].

Our findings suggest a potential role of M2-like monocytes and CD8^+^CD20^+^ T-cells as biomarkers for early detection and therapeutic targets in HIV-positive pre-NHL.

### 4.4. Immune Cell Correlations for HIV-Positive Pre-NHL

AICDA, an enzyme critical for somatic hypermutation and class-switch recombination during B-cell activation, plays a pivotal role in generating high-affinity antibodies in response to pathogens [60,61]. However, its dysregulated expression in B-cells may contribute to immune dysfunction and increase the risk of malignant transformation. Similarly, cMYC overexpression in B-cells is often associated with malignancy [9]. Hyperactivated B-cells may contribute to immune dysfunction and increase the risk of malignant transformation due to AICDA and cMYC expression. To further explore this hypothesis, we examined associations between previously identified AICDA^+^ B-cell populations in HIV-positive pre-NHL individuals [49].

In HIV-positive pre-NHL, we observed several correlations between specific immune cell populations that suggest a potential path toward malignancy. First, CD19^+^ B-cells expressing AICDA and cMYC (MC14) [49] were positively correlated with M1-like CD14^+^ monocytes, which also expressed cMYC (MC02). A second cluster of CD19^+^ B-cells expressing FoxP3, AICDA, and cMYC (MC21) [49] was positively linked to potentially exhausted CD8^+^PD-1^+^ T-cells (MC03).

The correlation of these B-cells with M1-like CD14^+^cMYC^+^ monocytes or CD8^+^PD-1^+^ T-cells may suggest that these immune cells play a synergistic role influencing a dysfunctional immune environment that could reduce effective immune responses against HIV and suppress the immune system’s ability to mount effective surveillance against pre-NHL B-cells. Moreover, M1-like CD14^+^cMYC^+^ monocytes or CD8^+^PD-1^+^ T-cells may be contributing to the immune activation and inflammation present in HIV infection that can contribute to lymphomagenesis.

### 4.5. CD8^+^PD-1^+^CD27^+^CXCR4^−^ T-Cells Positively Correlate with CD4^+^FoxP3^+^PD-1^+^ T-Cells in HIV-Positive Pre-NHL

In HIV-positive individuals at risk for developing non-Hodgkin lymphoma (pre-NHL), CD8^+^PD-1^+^CD27^+^CXCR4^−^ T-cells positively correlate with CD4^+^FoxP3^+^PD-1^+^ T-cells expressing CD27, CD28, ICOS, and CD71. The co-expression of PD-1 and CD27 suggests that these CD8^+^ T-cells are in a state of prolonged immune activation, characteristic of chronic HIV infection. While PD-1 indicates exhaustion, CD27 implies these T-cells retain some activation potential.

CD4^+^FoxP3^+^PD-1^+^ Tregs, known for their suppressive function, may regulate excessive immune activation by dampening immune responses. The elevated proportion of CD4^+^FoxP3^+^PD-1^+^ Tregs in HIV-positive pre-NHL individuals likely reflects an adaptive response to chronic inflammation but may also serve as a mechanism that hampers effective HIV control, increasing the risk of lymphoma development in pre-NHL states.

### 4.6. CD8^+^PD-1^+^CD27^+^CXCR4^−^ T-Cells Are Inversely Correlated with M2-like CD14^+^CD86^+^CD163^+^CXCR4^+^ Monocytes in HIV-Positive Pre-NHL

In HIV-positive individuals at risk for developing NHL (pre-NHL), CD8^+^PD-1^+^CD27^+^CXCR4^−^ T-cells negatively correlate with M2-like CD14^+^CD86^+^CD163^+^CXCR4^+^ monocytes, suggesting an inverse relationship. The presence of CXCR4 on these monocytes facilitates their trafficking to lymphoid tissues. CXCR4 also serves as a co-receptor for HIV entry, making it a potential target for HIV infection [36,37,42,44,62,63]. In chronic HIV infection, T-cell exhaustion limits the ability of CD8^+^ T-cells to clear HIV-infected cells, leading to persistent viral replication and immune dysfunction [62]. This immune shift from pro-inflammatory to immunosuppressive conditions could allow immune escape, enabling pre-tumor cells to evade surveillance and potentially promote the early developmental stages of lymphoma.

### 4.7. CD8^+^CD14^+^ T-Cells Positively Correlate with CD19^+^CD24^hi^CD38^hi^ Bregs in HIV-Positive Pre-NHL

In HIV-positive pre-NHL, CD8^+^CD14^+^ T-cells correlated with AICDA^+^ Bregs and IL-10^+^ Bregs. In HIV-positive cART-naïve individuals, we observed associations involving Bregs with CD8^+^PD-1^+^ T-cells expressing CD27, CXCR4, and CCR5. Elevated levels of Bregs in HIV infection play a role in modulating immune responses, potentially influencing disease progression and the development of HIV-associated malignancies [49,52,64,65,66,67]. The positive correlation between CD8^+^CD14^+^ T-cells and Bregs in both HIV-positive cART-naïve and HIV-positive pre-NHL suggests that these cells play a role in immune regulation in HIV and could potentially interact with each other. These results suggest that CD8^+^CD14^+^ T-cells and Bregs may work together to shape the immunosuppressive environment during HIV infection, warranting deeper investigation into their interplay and how they respond to LPS and microbial translocation.

Bregs are key modulators of immune homeostasis, primarily through their secretion of IL-10 and TGF-β, which help maintain tolerance and limit excessive inflammation. In the context of HIV infection, however, Breg activity may dampen protective immune responses, potentially hindering viral clearance. The association between AICDA^+^ Bregs and CD8^+^CD14^+^ T-cells raises the possibility that this T-cell population could contribute to the expansion or function of pre-tumorigenic B-cells expressing AICDA, highlighting a complex immunoregulatory axis that may influence both viral pathogenesis and B-cell dysregulation. Studies have shown that CD8^+^CD14^+^ T-cells accumulate in liver allografts and during hepatic viral and tumor-specific responses in a mouse model, producing IL-10 and IL-2 ex vivo [21]. Additionally, LPS can increase the production of CD8^+^CD14^+^ T-cells in vitro and in vivo, suggesting that microbial products in the gut-liver axis can impact CD8^+^ T-cell-mediated immune responses [21].

Bregs, CD8^+^CD20^+^ T-cells, CD8^+^CD14^+^ T-cells, and M2-like CD14^+^CD163^+^ monocytes may serve as early indicators of immune dysfunction in PWH at risk for developing AIDS-NHL, potentially months to years before diagnosis, as this cohort study suggests. To better understand their role in AIDS-NHL pathogenesis, future studies should examine the interactions between these B-cells, T-cells, and M2-like monocytes. Functional assays and longitudinal studies can provide insight into how these subsets contribute to tumor progression, comparing pre-NHL individuals before and after cART treatment, and after NHL diagnosis. Research should also explore how CD8^+^CD20^+^ T-cells or CD8^+^CD14^+^ T-cells interact with other immune cells in the pre-lymphoma microenvironment. Additionally, studying their role in secreting extracellular vesicles carrying CD20 or microbial translocation markers (e.g., LPS) will help clarify how interactions with B-cells contribute to the pre-lymphoma microenvironment and enter peripheral blood circulation.

HIV infection significantly accelerates chronic inflammatory states that facilitate malignant B-cell transformation, including immunosuppression that facilitates reactivation of EBV (associated with CNS and Hodgkin/non-Hodgkin lymphomas), human herpesvirus 8 (HHV-8) (driving primary effusion lymphomas (PEL)), and CMV (contributing to immunosenescence) [2,68,69]. Future studies will examine the tumor microenvironment of AIDS-NHL, EBV status, and tumor subtypes of NHL.

## 5. Limitations of the Study

This study analyzed PBMC samples from HIV-positive cART-naïve individuals who later developed NHL, specifically from visits conducted by participants of the Multicenter AIDS Cohort Study (MACS) between 1985 and 2002. It is important to note that the MACS study cohort primarily consisted of homosexual and bisexual men. Future studies can expand to other populations to capture the full range of immune responses and disease progression across different racial and ethnic demographics, including women. Expanding the cohort analysis would provide a more inclusive understanding of immune dysregulation in other HIV-associated malignancies, highlighting potential variations in disease progression and responses to therapy among different groups. This approach will ensure broader findings that can guide healthcare solutions for HIV-associated cancer treatment and for global HIV populations, where co-infections with EBV and KSHV are also common.

Our studies are also limited by small sample size for the HIV-positive pre-NHL group and the inability to conduct functional and mechanistic studies due to exhaustion of cell samples from the same cohort used in this study. MWCCS participant samples allowed us to reasonably draw from phenotypic data acquired from each cohort group. Future studies will include larger cohorts, fresh PBMCs, and data from functional assays to provide mechanistic insight into the malignant transformation of B-cells and immune cell responses in AIDS-NHL.

Lastly, without additional monocyte markers (CD68, CD33) in our CyTOF panel, we cannot definitively exclude monocyte–T-cell complexes or trogocytosis contributing to the CD8^+^CD14^+^ population identified in our studies. However, this is unlikely given the following: (1) consistent CD163 negativity across samples, and (2) peripheral blood sampling (trogocytosis predominates in tissues). CD8^+^CD14^+^ T-cells are well-documented in inflammatory conditions and LPS-inducible [21], supporting their biological plausibility in HIV infection and pre-AIDS-NHL, where LPS levels are elevated.

## 6. Conclusions

Through unsupervised clustering and immunophenotyping, we identified phenotypic alterations in circulating T-cells and monocytes in HIV-positive cART-naïve individuals at 3 to 36 months before NHL diagnosis. Our findings revealed a distinct subset of T-cells and monocytes with aberrant markers and oncogenic features, suggesting early immune changes that may contribute to lymphoma development in PWH.

Collectively, our analysis revealed significantly elevated cMYC^+^ AICDA^+^ B-cells, CD8^+^CD20^+^ T-cells, CD8^+^CD14^+^ T-cells, and M2-like CD14^+^CD163^+^ monocytes in HIV-positive pre-NHL cases (cART-naïve) compared to HIV-negative and HIV-positive cART-naïve cases, indicating critical dysregulation in immune cell homeostasis. Significant positive correlations were observed between CD19^+^AICDA^+^cMYC^+^ B-cells and M1-like CD14^+^cMYC^+^ monocytes (MC02), as well as between CD8^+^PD-1^+^CD27^+^CXCR4^−^ T-cells (MC05) and CD4^+^FoxP3^+^PD-1^+^CD27^+^CD28^+^CXCR4^−^ICOS^+^ T-cells (MC08) in HIV-positive pre-NHL cases. A distinct CD19^+^FoxP3^+^AICDA^+^cMYC^+^ B-cell cluster was also linked to CD8^+^PD-1^+^CD27^+^CD28^+^CXCR4^+^ T-cells (MC03). In contrast, CD8^+^PD-1^+^CD27^+^CXCR4^−^ T-cells (MC05) negatively correlated with M2-like CD14^+^CD163^+^ monocytes (MC06). Our studies found a positive correlation between CD8^+^CD14^+^ T-cells and AICDA^+^ and IL-10^+^ B-regs in HIV-positive pre-NHL individuals. These phenotypic signatures reflect continuous immune activation driving T-cell exhaustion and aberrant B-cell responses through AICDA-mediated somatic hypermutation.

In the context of chronic HIV infection, persistent inflammation and cytokine dysregulation create a perfect storm for lymphomagenesis by promoting clonal evolution under selective pressure, impairing immune surveillance mechanisms that normally suppress tumor development, and accelerating immune aging. These effects persist in PWH despite effective antiretroviral therapy. Our findings help interpret our current understanding of cell biomarkers in peripheral blood circulation of PWH and provide preliminary insight into the compressed timeline from immune dysfunction to pre-malignant transformation, particularly in HIV-positive cART-naïve individuals, where these processes are amplified and accelerated.

The scope of this study and our previous work [49] was to characterize B-cells with malignant phenotypes, as well as T-cells and monocytes, from HIV-positive pre-AIDS-NHL cases who were not on cART, in order to define early immune changes that occur prior to lymphoma development independent of cART effects. The direct comparisons were samples from HIV-positive cases that did not develop AIDS-NHL and HIV-negative controls. In future studies, we aim to compare PWH who develop AIDS-NHL with those who do not, examining samples collected before and after cART initiation in both groups.

By understanding early immune signatures in HIV-positive pre-NHL, we can pave the way for more targeted therapies and improved diagnostic strategies to reduce the incidence of lymphoma in PWH.

## Data Availability

The original contributions presented in the study are included in the article/Supplementary Materials. Further inquiries can be directed to the corresponding author.

## Supplementary Materials

The following supporting information can be downloaded at: https://www.mdpi.com/article/10.3390/cells14201608/s1, Table S1. Mass cytometry panel and source of metal-conjugated antibodies; Table S2. Related to Figure 1. Phenotypes of CD3^+^ T-cell (CD3^+^CD19^−^) metaclusters in HIV-negative, HIV-positive cART-naïve, and HIV-positive pre-NHL (cART-naïve), and significant differences in marker expression; Table S3. Related to Figure 3. Phenotypes of CD14^+^ monocyte (CD14^+^CD3^−^CD19^−^) metaclusters in HIV-negative, HIV-positive cART-naïve, and HIV-positive pre-NHL (cART-naïve), and significant differences in marker expression; Table S4. Related to Figure 4. Spearman correlation comparison between CD19^+^ B-cell metaclusters and CD3^+^ T-cells or CD19^+^ B-cell metaclusters and CD14^+^ monocyte metaclusters identified by unsupervised clustering analysis; Figure S1. CyTOF gating strategy and characterization of CD3^+^ T-cells (CD3^+^CD19^−^); Figure S2. Histogram plots of markers expressed on CD3^+^ T-cells (CD3^+^CD19^−^); Figure S3. UMAPs of CD4^+^ and CD8^+^ T-cells show expansion of CD8^+^ T-cells in HIV-positive cART-naïve and HIV-positive pre-NHL (cART-naïve) compared with HIV-negative; Figure S4. Total count of CD3^+^ T-cell (CD3^+^CD19^−^) metaclusters; Figure S5. Gating strategy of CD8^+^CD14^+^ monocytes (CD3^+^CD8^+^CD14^+^); Figure S6. Gating strategy of CD14^+^ monocytes (CD14^+^CD3^−^CD19^−^); Figure S7. Histogram plots of markers expressed on CD14^+^ monocytes (CD14^+^CD3^−^CD19^−^); Figure S8. Total count of CD14^+^ monocyte (CD14^+^CD3^−^CD19^−^) metaclusters.
