# Supplementary material for: Description of T-Cell and Monocyte Populations in the Circulation of People with HIV Prior to AIDS-NHL Diagnosis
Source: Cells. 2025 Oct 16;14(20):1608. doi: 10.3390/cells14201608 (PMC12564559; doi:10.3390/cells14201608)
Supplement: Supplementary file 1 [file cells-14-01608-s001.zip › cells-3877604-supplementary.pdf]

Supplementary Material

Supplementary Table S1. Mass cytometry panel and source of metal-conjugated antibodies.

Metal Label	Marker	Clone	Company Supplier	Catalog Number
**Surface panel**				
^{89}Y	CD3	UCHT1	BioLegend[a]	317302
^{106}Cd	CD14	M5E2	BioLegend	301810
^{110}Cd	HLA-DR	L243	BioLegend	307648
^{111}Cd	CD4	RPA-T4	BioLegend	300502
^{113}Cd	CD8a	RPA-T8	BioLegend	301002
^{114}Cd	IgG Fc	N/A	Jackson ImmunoResearch Labs[b]	AB_2337530
^{116}Cd	CD20	2H7	BioLegend	302302
^{141}Pr	IgM	MHM-88	BioLegend	314502
^{142}Nd	CD278 (ICOS)	C398.4A	BioLegend	313512
^{143}Nd	CD183 (CXCR3)	G025H7	BioLegend	353750
^{144}Nd	CD195 (CCR5)	NP-6G4	Fluidigm/Standard BioTools[c]	3144007A
^{145}Nd	CD163	GHI/61	BioLegend	333602
^{146}Nd	CD10	HI10a	BioLegend	312202
^{147}Sm	CD24	ML5	BioLegend	311127
^{149}Sm	CD19	HIB19	BioLegend	302214
^{150}Nd	CD86	IT2.2	Fluidigm/Standard BioTools	3150020B
^{151}Eu	Ig lambda (light chain)	MHL-38	Fluidigm/Standard BioTools	3151004B
^{153}Eu	CD185 (CXCR5)	RF8B2	Fluidigm/Standard BioTools	3142015B
^{155}Gd	CD273 (PD-L2)	24F.10C12	BioLegend	329610
^{156}Gd	CD184 (CXCR4)	12G5	BioLegend	306512
^{158}Gd	CD27	O323	Fluidigm/Standard BioTools	302802
^{159}Tb	CD284 (TLR4)	HTA125	BioLegend	312808
^{160}Gd	Ig kappa (light chain)	MHK-49	Fluidigm/Standard BioTools	3160005B
^{164}Dy	CD38	HIT2	BioLegend	303502

^{165}Ho	CD40	5C3	Fluidigm/Standard BioTools	3165005B
^{168}Er	CD154 (CD40L)	24-31	Fluidigm/Standard BioTools	3168006B
^{170}Er	CD152 (CTLA-4)	14D3	Invitrogen[d]	3170005B
^{171}Yb	CD28	CD28.2	BioLegend	359602
^{172}Yb	CD279 (PD-1)	EH12.2H7	BioLegend	329912
^{173}Yb	CD80	2D10.4	BioLegend	305246
^{174}Yb	CD71	OKT	Invitrogen	14-0719-82
^{175}Lu	CD274 (PD-L1)	29E.2A3	BioLegend	329716
^{209}Bi	CD11b	ICRF44	Fluidigm/Standard BioTools	3209003B
**Intracellular panel**				
^{162}Dy	FoxP3	PCH101	Fluidigm/Standard BioTools	3162011A
^{163}Dy	Bcl-6	K112-91	Fluidigm/Standard BioTools	3163012B
^{166}Er	IL-10	JES3-9D7	Fluidigm/Standard BioTools	3166008B
^{169}Tm	AICDA	359218	R&D Systems[e]	MAB39102
^{176}Yb	c-MYC	9.00E+10	Fluidigm/Standard BioTools	3176012B
^{152}Sm	EBV LMP1	LMPO24	Novus Biologicals[f]	NBP2-50383
^{154}Sm	HIV-1 core antigen	KC57	Beckman Coulter[g]	IMBULK1B

[a]BioLegend, San Diego, CA, USA; [b]Jackson ImmunoResearch Labs, West Grove, PA, USA; [c]Fluidigm/Standard BioTools, South San Francisco, CA, USA; [d]Invitrogen, Waltham, MA, USA; [e]R&D Systems, Minneapolis, MN, USA; [f]Novus Biologicals, Centennial, CO, USA; [g]Beckman Coulter, Brea, CA.

Supplementary Table S2. Related to Figure 1. Phenotypes of CD3$^+$ T-cell (CD3$^+$CD19$^-$) metaclusters in HIV-negative, HIV-positive cART-naïve, and HIV-positive pre-NHL (cART-naïve), and significant differences in marker expression.

**Metacluster**[a]	**Sample**	**Phenotype**	**Marker expression**	***p*-value**[b]
MC01	HIV-negative	**CD4**$^+$ CD27$^+$ CD28$^+$ CXCR4$^+$	CD4 elevated in HIV-negative vs. HIV-positive cART-naïve	$p = 0.026$
	HIV-positive cART-naïve	CD4$^+$ CD27$^+$ CD28$^+$ CXCR4$^+$		
	HIV-positive pre-NHL (cART-naïve)	CD4$^+$ CD27$^+$ CD28$^+$ CXCR4$^+$		
MC02	HIV-negative[c]	**CD4**$^+$ CD8$^+$ CXCR4$^+$	CD4 elevated in HIV-negative vs. HIV-positive cART-naïve<br><br>CD4 elevated in HIV-negative vs. HIV-positive pre-NHL	$p = 0.026$<br><br>$p = 0.012$
	HIV-positive cART-naïve	CD4$^+$ CD8$^+$ CXCR4$^+$		
	HIV-positive pre-NHL (cART-naïve)	CD4$^+$ CD8$^+$ CXCR4$^+$ HLA-DR$^+$		
MC03	HIV-negative	**CD8**$^+$ CD27$^+$ **CD28**$^+$ CXCR4$^+$	CD8 elevated in HIV-negative vs. HIV-positive cART-naïve<br><br>CD8 elevated in HIV-negative vs. HIV-positive pre-NHL<br><br>CD28 elevated in HIV-negative vs. HIV-positive pre-NHL	$p = 0.012$<br><br>$p = 0.004$<br><br>$p = 0.011$
	HIV-positive cART-naïve	CD8$^+$ CD27$^+$ CD28$^+$ CXCR4$^+$		
	HIV-positive pre-NHL (cART-naïve)	CD8$^+$ CD27$^+$ CD28$^+$ CXCR4$^+$ **PD-1**$^+$	PD-1 elevated in HIV-positive pre-NHL vs. HIV-negative	$p = 0.017$

MC04	HIV-negative	$CD4^{+}$ $CD8^{+}$ $CD27^{+}$ **$CD28^{+}$ $CXCR4^{+}$ $FoxP3^{+}$**	FoxP3 elevated in HIV-negative vs. HIV-positive cART-naïve<br><br>CXCR4 elevated in HIV-negative vs. HIV-positive cART-naïve<br><br>CD28 elevated in HIV-negative vs. HIV-positive cART-naïve<br><br>CD28 elevated in HIV-negative vs. HIV-positive pre-NHL	*p* = 0.012<br><br>*p* = 0.002<br><br>*p* = 0.047<br><br>*p* = 0.031
	HIV-positive cART-naïve	$CD4^{+}$ $CD8^{+}$ $CD27^{+}$ $CD28^{+}$ $CXCR4^{+}$ $FoxP3^{-}$		
	HIV-positive pre-NHL (cART-naïve)	$CD4^{+}$ $CD8^{+}$ $CD27^{+}$ $CD28^{+}$ $CXCR4^{+}$ $FoxP3^{+}$		
MC05	HIV-negative	**$CD8^{+}$** $CD27^{+}$ $CXCR4^{+}$	CD8 elevated in HIV-negative vs. HIV-positive cART-naïve<br><br>CD8 elevated in HIV-negative vs. HIV-positive pre-NHL	*p* = 0.021<br><br>*p* = 0.004
	HIV-positive cART-naïve	$CD8^{+}$ $CD27^{+}$ $CXCR4^{+}$ $CCR5^{+}$ **$PD\text{-}1^{+}$ $HLA\text{-}DR^{+}$**	PD-1 elevated in HIV-positive cART-naïve vs. HIV-negative<br><br>HLA-DR elevated in HIV-positive cART-naïve vs. HIV-negative	*p* = 0.034<br><br>*p* = 0.027
	HIV-positive pre-NHL (cART-naïve)	$CD8^{+}$ $CD27^{+}$ $CXCR4^{-}$ **$PD\text{-}1^{+}$** $HLA\text{-}DR^{+}$	PD-1 elevated in HIV-positive pre-NHL vs. HIV-negative	*p* = 0.015
MC06	HIV-negative	$CD4^{+}$ $CD20^{-}$ $CD27^{+}$ $CD28^{+}$ **$CXCR4^{+}$** $CD14^{-}$ $CD11b^{+}$ $HLA\text{-}DR^{+}$	CXCR4 elevated in HIV-negative vs. HIV-positive cART-naïve	*p* = 0.007
	HIV-positive cART-naïve	$CD4^{+}$ $CD20^{+}$ $CD27^{+}$ $CD28^{+}$ $CXCR4^{+}$ $CD14^{+}$ $CD11b^{+}$ $HLA\text{-}DR^{+}$		
	HIV-positive pre-NHL (cART-naïve)	$CD4^{+}$ $CD20^{+}$ $CD27^{+}$ $CD28^{+}$ $CXCR4^{+}$ $CD14^{-}$ $CD11b^{+}$ $HLA\text{-}DR^{+}$		
MC07	HIV-negative	$CD8^{+}$ $CD27^{-}$ $CXCR4^{+}$	Potential complex with $CD163^{+}$ monocytes	

	HIV-positive cART-naïve	CD8^{+} CD27^{-} CXCR4^{+} CD163^{+}		
	HIV-positive pre-NHL (cART-naïve)	CD8^{+} CD27^{+} CXCR4^{+} CD163^{+}		
MC08	HIV-negative	CD4^{+} CD27^{+} CD28^{+} CXCR4^{+} ICOS^{+} **FoxP3^{+}** PD-1^{+} CD71^{+} HLA-DR^{+}	FoxP3 elevated in HIV-negative vs. HIV-positive cART-naïve	$p = 0.013$
	HIV-positive cART-naïve	CD4^{+} CD27^{+} CD28^{+} CXCR4^{+} ICOS^{+} FoxP3^{-} PD-1^{+} CD71^{+} HLA-DR^{+}		
	HIV-positive pre-NHL (cART-naïve)	CD4^{+} CD27^{+} CD28^{+} CXCR4^{-} ICOS^{+} **FoxP3^{+}** PD-1^{+} CD71^{+} HLA-DR^{+}	FoxP3 elevated in HIV-positive pre-NHL vs. HIV-positive	$p = 0.015$
MC09	HIV-negative	CD8^{+} CD20^{+} CD27^{+} **CD28^{+} CXCR4^{+}** CCR5^{+} CD11b^{+} HLA-DR^{+}	CXCR4 elevated in HIV-negative vs. HIV-positive<br><br>CD28 elevated in HIV-negative vs. HIV-positive pre-NHL	$p = 0.008$<br><br>$p = 0.033$
	HIV-positive cART-naïve	CD4^{+} CD8^{+} CD20^{+} CD27^{+} CD28^{-} CXCR4^{+} CCR5^{-} PD-1^{+} CD11b^{+} HLA-DR^{+}		
	HIV-positive pre-NHL (cART-naïve)	CD8^{+} CD20^{+} CD27^{+} CD28^{-} CXCR4^{-} CCR5^{+} PD-1^{+} CD11b^{+} HLA-DR^{+}		
MC10	HIV-negative	CD4^{+} CD8^{-} CD20^{-} CXCR4^{+} CD14^{+} CD11b^{+} HLA-DR^{+}		
	HIV-positive cART-naïve	CD4^{+} **CD8^{+} CD20^{+}** CXCR4^{+} CD14^{+} CD11b^{+} HLA-DR^{+}	CD8 elevated in HIV-positive vs. HIV-positive pre-NHL<br><br>CD20 elevated in HIV-positive vs. HIV-negative	$p = 0.003$<br><br>$p = 0.007$
	HIV-positive pre-NHL (cART-naïve)	CD4^{+} CD8^{-} CD20^{+} CXCR4^{+} CD14^{+} CD11b^{+} HLA-DR^{+}		
MC11	HIV-negative	CD4^{+} CXCR4^{+}		
	HIV-positive cART-naïve	CD4^{+} CXCR4^{+} **HLA-DR^{+}**	HLA-DR elevated in HIV-positive cART-naïve vs. HIV-negative	$p < 0.0001$
	HIV-positive pre-NHL (cART-naïve)	CD4^{+} CXCR4^{-} CD28^{+} PD-1^{+} **HLA-DR^{+}**	HLA-DR elevated in HIV-positive pre-NHL vs. HIV-negative	$p = 0.004$

MC12	HIV-negative	CD3^{+} CD4^{-} CD8^{-} CXCR4^{+}		
	HIV-positive cART-naïve	CD3^{+} CD4^{-} CD8^{-} CXCR4^{+} **HLA-DR^{+}**	HLA-DR elevated in HIV-positive cART-naïve vs. HIV-negative	$p = 0.004$
	HIV-positive pre-NHL (cART-naïve)	CD3^{+} CD4^{-} CD8^{-} CXCR4^{+} **HLA-DR^{+}**	HLA-DR elevated in HIV-positive pre-NHL vs. HIV-negative	$p = 0.029$
MC13	HIV-negative	CD8^{+} CXCR4^{+}		
	HIV-positive cART-naïve	CD8^{+} CXCR4^{+} **HLA-DR^{+}**	HLA-DR elevated in HIV-positive cART-naïve vs. HIV-negative<br><br>HLA-DR elevated in HIV-positive cART-naïve vs. HIV-positive pre-NHL	$p < 0.0001$<br><br>$p = 0.022$
	HIV-positive pre-NHL (cART-naïve)	CD8^{+} CXCR4^{-}		
MC14	HIV-negative	CD8^{+} CD20^{-} **CXCR4^{+}** CCR5^{+} CD11b^{+} HLA-DR^{+}	CXCR4 elevated in HIV-negative vs. HIV-positive cART-naïve	$p = 0.031$
	HIV-positive cART-naïve	**CD4^{+}** CD8^{+} CD20^{+} CXCR4^{-} CCR5^{-} CD11b^{+} HLA-DR^{+}	CD4 elevated in HIV-positive cART-naïve vs. HIV-positive pre-NHL	$p = 0.041$
	HIV-positive pre-NHL (cART-naïve)	CD8^{+} CD20^{+} CXCR4^{-} CCR5^{+} CD11b^{+} HLA-DR^{+}		
MC15	HIV-negative	CD4^{+} CD20^{-} CD27^{+} CXCR4^{+} FoxP3^{+} BCL-6^{+} IL-10^{+} CXCR5^{+} CXCR3^{+} cMYC^{+} CD14^{+} CD86^{+} CD11b^{+} HLA-DR^{+}		
	HIV-positive cART-naïve	CD4^{+} CD8^{+} CD20^{+} CD27^{-} CXCR4^{+} FoxP3^{+} BCL-6^{+} cMYC^{+} CD14^{+} CD86^{+} CD11b^{+} HLA-DR^{+}		
	HIV-positive pre-NHL (cART-naïve)	CD4^{+} CD8^{+} CD20^{+} CD27^{-} CXCR4^{+} CCR5^{+} FoxP3^{+} BCL-6^{+} cMYC^{+} CD14^{+} CD86^{+} CD11b^{+} HLA-DR^{+}		

Supplementary Table S3. Related to Figure 3. Phenotypes of CD14^{+} monocyte (CD14^{+}CD3^{-}CD19^{-}) metaclusters in HIV-negative, HIV-positive cART-naïve, and HIV-positive pre-NHL (cART-naïve), and significant differences in marker expression**.**

**Metacluster**[a]	**Sample**	**Phenotype**	**Marker expression**	***p*-value**[b]
MC01	HIV-negative	CD14^{+} HLA-DR^{+} CD11b^{+} CD4^{+} **CXCR4^{+}**	CXCR4 elevated in HIV-negative vs. HIV-positive cART-naïve<br><br>CXCR4 elevated in HIV-negative vs. HIV-positive pre-NHL	$p = 0.0003$<br><br>$p = 0.050$
	HIV-positive cART-naïve	CD14^{+} HLA-DR^{+} CD11b^{+} CD4^{+} CXCR4^{-}		
	HIV-positive pre-NHL (cART-naïve)	CD14^{+} HLA-DR^{+} CD11b^{+} CD4^{+} CXCR4^{-}		
MC02	HIV-negative	CD14^{+} CD86^{+} HLA-DR^{+} CD11b^{+} CD4^{+} **CXCR4^{+}** cMYC^{+}	CXCR4 elevated in HIV-negative vs HIV-positive cART-naïve	$p = 0.013$
	HIV-positive cART-naïve	CD14^{+} CD86^{+} **HLA-DR^{+}** CD11b^{+} CD4^{+} CXCR4^{+} cMYC^{-}	HLA-DR elevated in HIV-positive cART-naïve vs. HIV-positive pre-NHL	$p = 0.017$
	HIV-positive pre-NHL (cART-naïve)	CD14^{+} CD86^{+} HLA-DR^{+} CD11b^{+} CD4^{+} CXCR4^{+} cMYC^{+}		
MC03	HIV-negative[c]	CD14^{+} CD86^{+} HLA-DR^{+} CD11b^{+} CD4^{+} FoxP3^{+} BCL-6^{+} IL-10^{+} CXCR4^{+} cMYC^{+}		
	HIV-positive cART-naïve	CD14^{+} CD86^{+} HLA-DR^{+} CD11b^{+} CD4^{+} FoxP3^{+} BCL-6^{+} IL-10^{+} CXCR4^{+} cMYC^{+}		
	HIV-positive pre-NHL (cART-naïve)	CD14^{+} CD86^{+} HLA-DR^{+} CD11b^{+} CD4^{+} FoxP3^{+} BCL-6^{+} IL-10^{+} CXCR4^{+} cMYC^{+}		
MC04	HIV-negative	CD14^{+} CD86^{+} CD163^{+} HLA-DR^{+} CD11b^{+} CD4^{+} FoxP3^{+} BCL-6^{+} CXCR4^{+} CCR5^{+} cMYC^{+}		
	HIV-positive cART-naïve	CD14^{+} CD86^{+} CD163^{+} HLA-DR^{+} CD11b^{+} CD4^{+} FoxP3^{+} BCL-6^{+} CXCR4^{+} CCR5^{+} cMYC^{+}		
	HIV-positive pre-NHL (cART-naïve)	CD14^{+} CD86^{+} CD163^{-} HLA-DR^{+} CD11b^{+} CD4^{+} FoxP3^{+} BCL-6^{-} CXCR4^{+} CCR5^{+} cMYC^{+}		

MC05	HIV-negative	CD14$^+$ HLA-DR$^+$ CD11b$^+$ CD4$^+$ **CXCR4$^+$**	CXCR4 elevated in HIV-negative vs HIV-positive cART-naïve	$p = 0.012$
	HIV-positive cART-naïve	CD14$^+$ HLA-DR$^+$ CD11b$^+$ CD4$^+$ CXCR4$^-$		
	HIV-positive pre-NHL (cART-naïve)	CD14$^+$ HLA-DR$^+$ CD11b$^+$ CD4$^+$ **CXCR4$^+$**	CXCR4 elevated in HIV-positive pre-NHL vs. HIV-positive cART-naïve	$p = 0.050$
MC06	HIV-negative	CD14$^+$ CD86$^-$ CD163$^+$ HLA-DR$^+$ CD11b$^+$ CD4$^+$ **CXCR4$^+$**	CXCR4 is elevated in HIV-negative vs. HIV-positive cART-naïve	$p = 0.027$
	HIV-positive cART-naïve	CD14$^+$ CD86$^+$ CD163$^+$ HLA-DR$^+$ CD11b$^+$ CD4$^+$ CXCR4$^+$ CCR5$^+$	CCR5 elevated in HIV-positive cART-naïve vs. HIV-negative	$p = 0.050$
	HIV-positive pre-NHL (cART-naïve)	CD14$^+$ CD86$^+$ CD163$^+$ HLA-DR$^+$ CD11b$^+$ CD4$^+$ CXCR4$^+$ CCR5$^+$	CCR5 elevated in HIV-positive pre-NHL vs. HIV-negative	$p = 0.050$
MC07	HIV-negative	CD14$^+$ HLA-DR$^+$ CD11b$^+$ CD4$^+$ **BCL-6$^+$** CXCR4$^+$	BLC-6 elevated in HIV-negative vs. HIV-positive cART-Naïve<br><br>BLC-6 elevated in HIV-negative vs. HIV-positive pre-NHL	$p = 0.001$<br><br>$p < 0.0001$
	HIV-positive cART-naïve	CD14$^+$ HLA-DR$^+$ CD11b$^+$ CD4$^+$ BCL-6$^-$ CXCR4$^+$		
	HIV-positive pre-NHL (cART-naïve)	CD14$^+$ HLA-DR$^+$ CD11b$^+$ CD4$^+$ BCL-6$^-$ CXCR4$^+$		
MC08	HIV-negative	CD14$^+$ HLA-DR$^+$ CD11b$^+$ CD4$^+$ **CXCR4$^+$**		
	HIV-positive cART-naïve	CD14$^+$ **HLA-DR$^+$** CD11b$^+$ CD4$^+$ CXCR4$^-$	HLA-DR elevated in HIV-positive cART-naïve vs. HIV-positive pre-NHL<br><br>CD4 elevated in HIV-positive cART-naïve vs. HIV-positive pre-NHL	$p = 0.037$<br><br>$p = 0.025$
	HIV-positive pre-NHL (cART-naïve)	CD14$^+$ HLA-DR$^+$ CD11b$^+$ CD4$^-$ CXCR4$^-$		
MC09	HIV-negative	CD14$^+$ HLA-DR$^+$ CD11b$^+$ CD4$^+$ **BCL-6$^+$** **CXCR4$^+$** cMYC$^+$	BCL-6 elevated in HIV-negative vs. HIV-positive cART-naïve	$p < 0.0001$

			BLC-6 elevated in HIV-negative vs. HIV-positive Pre-NHL<br><br>CXCR4 elevated in HIV-negative vs. HIV-positive cART-naïve<br><br>CXCR4 elevated in HIV-negative vs HIV-positive pre-NHL	$p = 0.0005$<br><br>$p = 0.0002$<br><br>$p = 0.005$
	HIV-positive cART-naïve	$CD14^+$ HLA-DR^+ $CD11b^+$ $CD4^+$ BCL-6^- $CXCR4^+$		
	HIV-positive pre-NHL (cART-naïve)	$CD14^+$ $CD86^+$ HLA-DR^+ $CD11b^+$ $CD4^+$ BCL-6^+ $CXCR4^+$ $cMYC^+$		
MC10	HIV-negative	$CD14^+$ $CD163^+$ HLA-DR^+ $CD11b^+$ $CD4^+$ $CXCR4^+$		
	HIV-positive cART-naïve	$CD14^+$ $CD163^+$ HLA-DR^+ $CD11b^+$ $CD4^+$ $CXCR4^+$		
	HIV-positive pre-NHL (cART-naïve)	$CD14^+$ $CD163^+$ HLA-DR^+ $CD11b^+$ $CD4^+$ $CXCR4^+$		

Supplementary Table S4. Related to Figure 4. Spearman correlation comparison between $CD19^{+}$ B-cell metaclusters and $CD3^{+}$ T-cells or $CD19^{+}$ B-cell metaclusters and $CD14^{+}$ monocyte metaclusters identified by unsupervised clustering analysis.

Spearman Correlation		95% Confidence Interval	Spearman r	*p*-value
**HIV-positive pre-NHL (cART-naïve) individuals**				
**$CD19^{+}$ B-cells, MC14**[a]<br><br>Phenotype:<br>$CD20^{+}$ $CD27^{+}$ $CD24^{+}$ $CD40^{+}$ $CXCR4^{+}$$CXCR5^{+}$$AICDA^{+}$$cMYC^{+}$	**$CD14^{+}$ monocytes, MC02**<br><br>Phenotype:<br>$CD14^{+}$ $CD86^{+}$ HLA-DR^{+} $CD11b^{+}$ $CD4^{+}$ $CXCR4^{+}$ $cMYC^{+}$	-0.010 to 0.900	0.761	0.018
**$CD19^{+}$ B-cells, MC21**[b]<br><br>Phenotype:<br>$CD20^{+}$ $CD27^{+}$ $CD24^{+}$ $CD40^{+}$ $CXCR4^{+}$ $CXCR5^{+}$ $FoxP3^{+}$ $AICDA^{+}$ $cMYC^{+}$	**$CD3^{+}$ T-cells, MC03**<br><br>Phenotype:<br>$CD8^{+}$ PD-1^{+} $CD27^{+}$ $CD28^{+}$ $CXCR4^{+}$	0.147 to 0.926	0.775	0.014
**$CD3^{+}$ T-cells, MC05**<br><br>Phenotype:<br>$CD8^{+}$ PD-1^{+} $CD27^{+}$ $CXCR4^{-}$ HLA-DR^{+}	**$CD3^{+}$ T-cells, MC08**<br><br>Phenotype:<br>$CD4^{+}$ $FoxP3^{+}$ PD-1^{+} $CD27^{+}$ $CD28^{+}$ $CXCR4^{-}$ $ICOS^{+}$ $CD71^{+}$ HLA-DR^{+}	0.573 to 0.972	0.879	0.002
	**$CD14^{+}$ monocytes, MC06**<br><br>Phenotype:<br>$CD14^{+}$ $CD86^{+}$ $CD163^{+}$ HLA-DR^{+} $CD11b^{+}$ $CD4^{+}$ $CXCR4^{+}$ $CCR5^{+}$	-0.587 to 0.669	-0.685	0.035
**$CD8^{+}$ $CD14^{+}$ T-cells**	**$AICDA^{+}$ Bregs ($CD19^{+}$ $CD24^{hi}$ $CD38^{hi}$)**	-0.602 to 0.656	0.800	0.008
	**IL-10^{+} Bregs ($CD19^{+}$ $CD24^{hi}$ $CD38^{hi}$)**	-0.612 to 0.646	0.708	0.027
**HIV-positive cART-naïve individuals**				
**$CD19^{+}$ $CD24^{hi}$ $CD38^{hi}$ Bregs**	**$CD3^{+}$ T-cells, MC05**<br><br>Phenotype:<br>$CD8^{+}$ PD-1^{+} $CD27^{+}$ $CXCR4^{+}$ $CCR5^{+}$ HLA-DR^{+}	0.401 to 0.887	0.723	0.0003

a. $CD19^{+}$ B-cell metacluster 14 identified and described in Martinez, L.E. *et al.* (2024). *Front. Immunol.* [49].

b. $CD19^{+}$ B-cell metacluster 21 identified and described in Martinez, L.E. *et al.* (2024). *Front. Immunol.* [49].

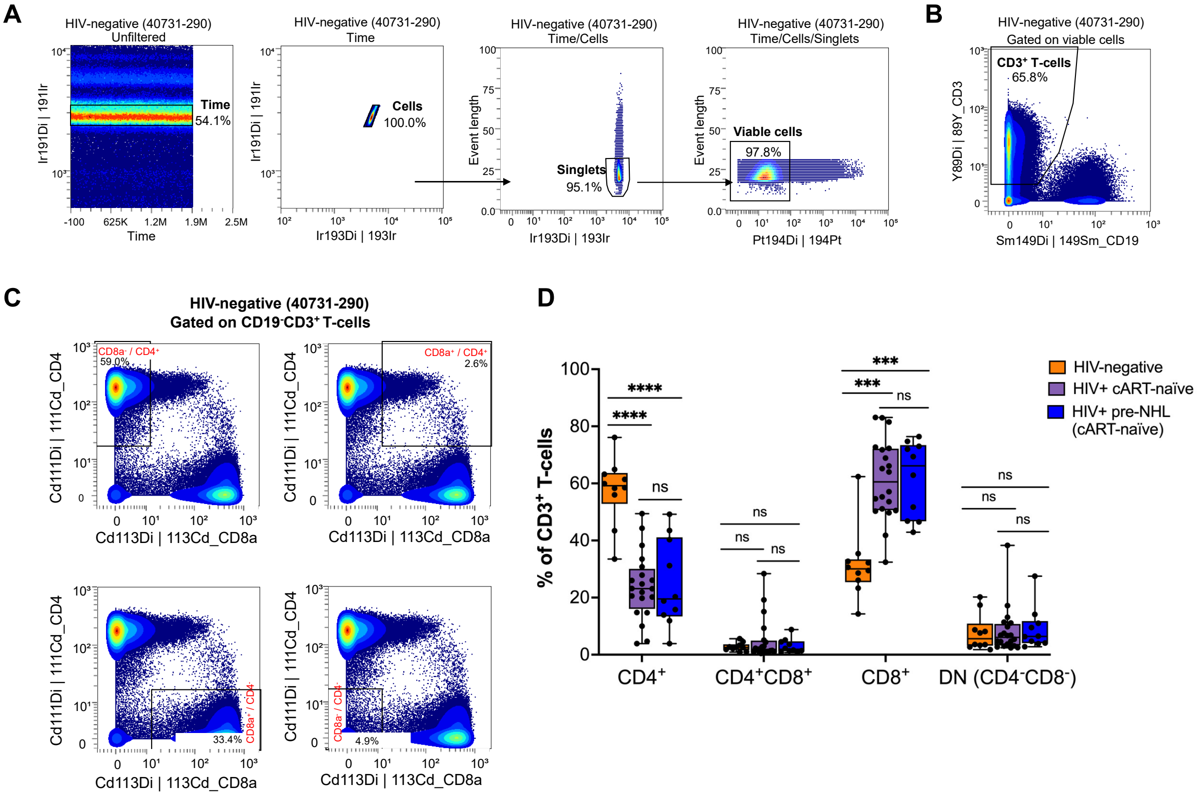

Supplementary Figure S1. CyTOF gating strategy and characterization of CD3$^+$ T-cells (CD3$^+$CD19$^-$). (**A**) Representative gating strategy of PBMCs after CyTOF. (**A**) Cell-ID Intercalator-Ir (191Ir) vs. time was used to select cells and gating of singlets.194Pt was used to discriminate live (viable) and dead cells. (**B**) Gating of CD3$^+$ T-cells from viable cells. (**C**) Gating strategy of CD3$^+$CD4$^+$ and CD3$^+$CD8$^+$ T-cells, double positive T-cells (CD3$^+$CD4$^+$CD8$^+$), CD3$^+$ double negative (DN, CD4$^-$CD8$^-$) T-cells. (**D**) Quantification and comparison in the percentage of CD3$^+$ T-cells (CD19$^-$) from total viable cells in PBMCs in HIV-negative (n = 10), HIV-positive (n = 20), and HIV-positive pre-NHL (n = 10). Mann-Whitney tests were performed to determine significance: *p*-values ≤ 0.05. ns = non-significant.

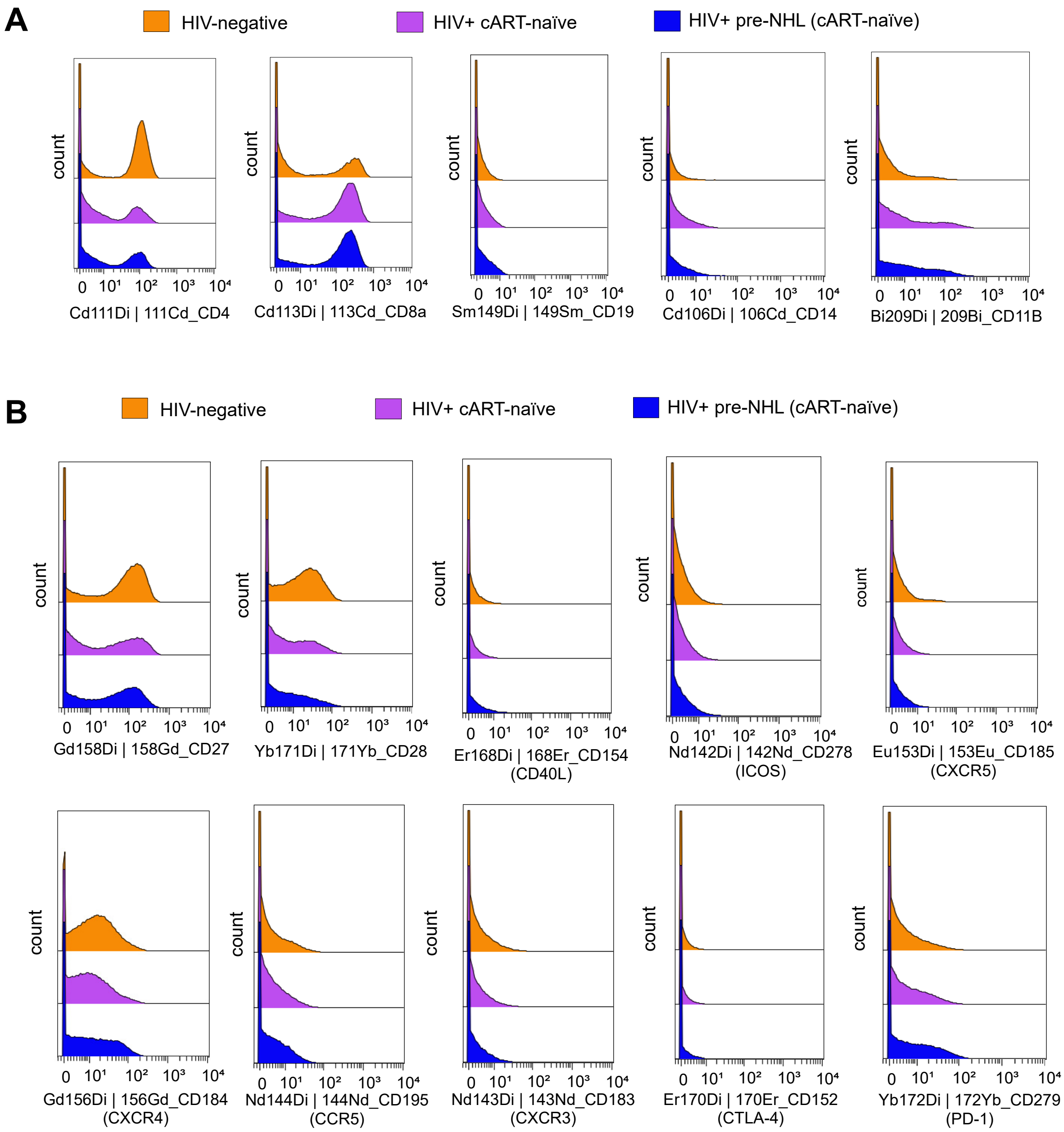

Supplementary Figure S2. Histogram plots of markers expressed on CD3^{+} T-cells (CD3^{+}CD19^{-}). (**A**) Surface marker expression of CD4, CD8, CD19, CD14, and CD11b in CD3^{+} T-cells (CD19^{-}) after CyTOF of PBMCs from HIV-negative (n = 10), HIV-positive cART-naïve (n = 20), and HIV-positive pre-NHL (cART-naïve) (n = 10) cohort participants. (**B**) Surface marker expression of select markers of T-cell activation (CD27, CD28, CD40L, ICOS), chemokine receptors (CXCR5, CXCR4, CCR5, CXCR3), and markers of exhaustion (CTLA-4, PD-1).

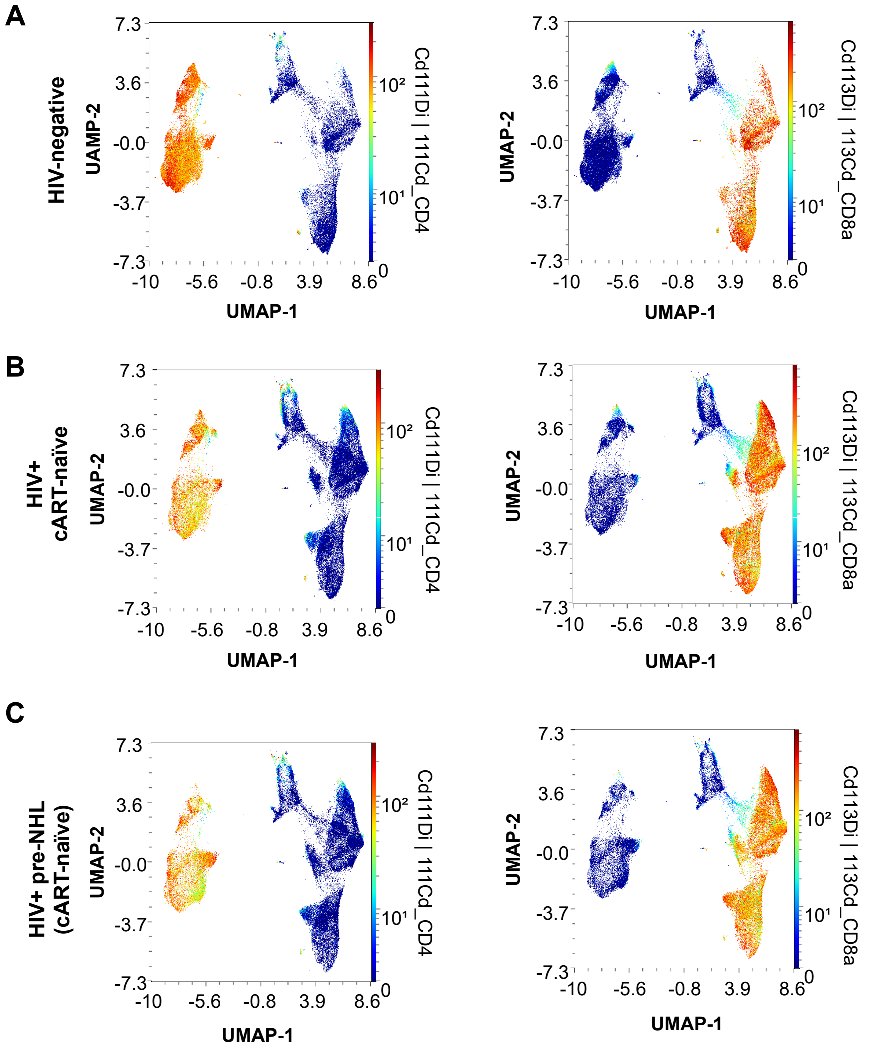

Supplementary Figure S3. UMAPs of CD4⁺ and CD8⁺ T-cells show expansion of CD8⁺ T-cells in HIV-positive cART-naïve and HIV-positive pre-NHL (cART-naïve) compared with HIV-negative. Contour plot and scatter plots of CD4 and CD8 marker expression in CD3⁺ T-cells for (**A**) HIV-negative (n =10), (**B**) HIV-positive cART-naïve (n = 20), and (**C**) HIV-positive pre-NHL (cART-naïve) (n = 10). UMAP plots were generated from an equal subsampling of 75,000 CD3⁺ T-cells (CD3⁺CD19⁻) as shown in **Figure 1A**.

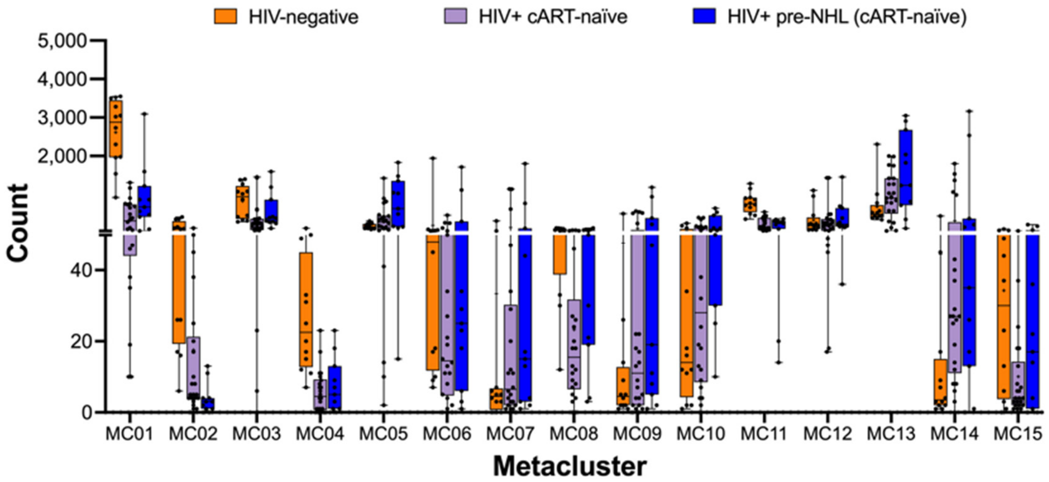

Supplementary Figure S4. Total count of CD3+ T-cell (CD3+CD19-) metaclusters. Box plots showing cell counts for each metacluster shown in **Figure 1A.**

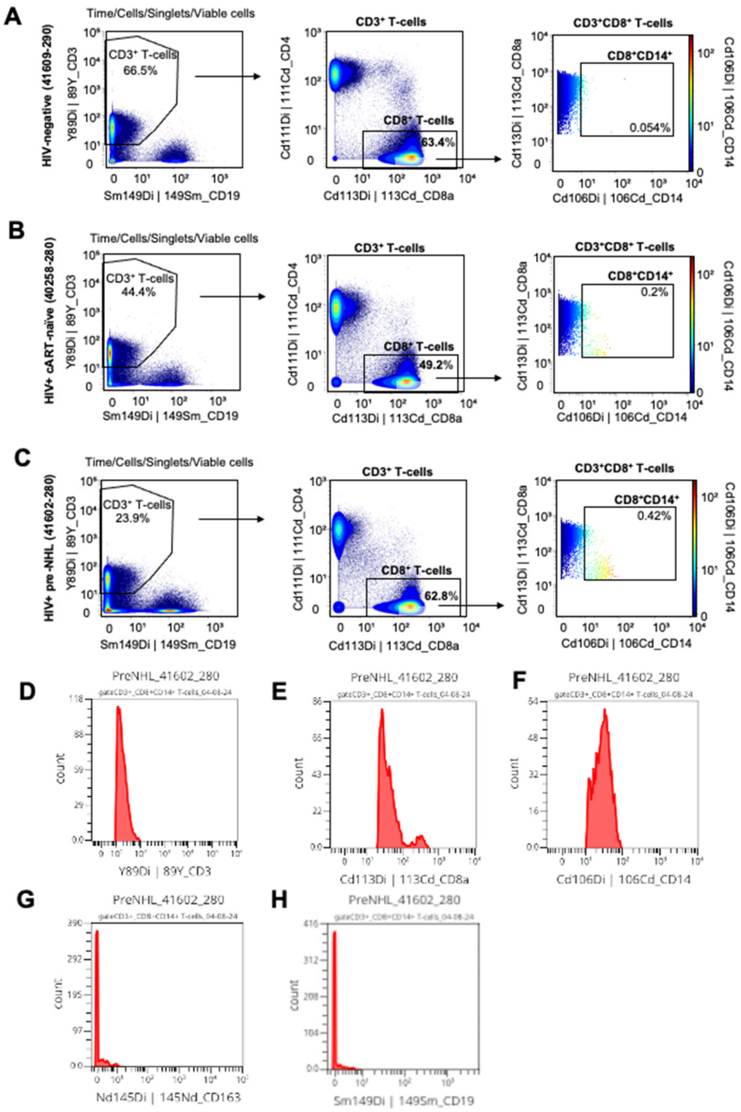

Supplementary Figure S5. Gating strategy of CD8⁺CD14⁺ monocytes (CD3⁺CD8⁺CD14⁺). Representative gating strategy of mass cytometry data to identify CD8⁺CD14⁺ T-cells from CD3⁺ CD8⁺ T-cells. Data shown is for an (**A**) HIV-negative sample (41609-290), (**B**) HIV-positive cART-naïve sample (40258-280), and (**C**) HIV-positive pre-NHL (cART-naïve) sample (41602-280). (**D-H**) Histogram plots of CD8⁺CD14⁺ T-cell subpopulation in an HIV-positive pre-NHL (cART-naïve) sample (41602-280) showing expression of CD3, CD8, CD14, CD163 and CD19. A representative HIV-positive pre-NHL (cART-naïve) sample is shown (41602-280); gating strategy was consistent across all groups.

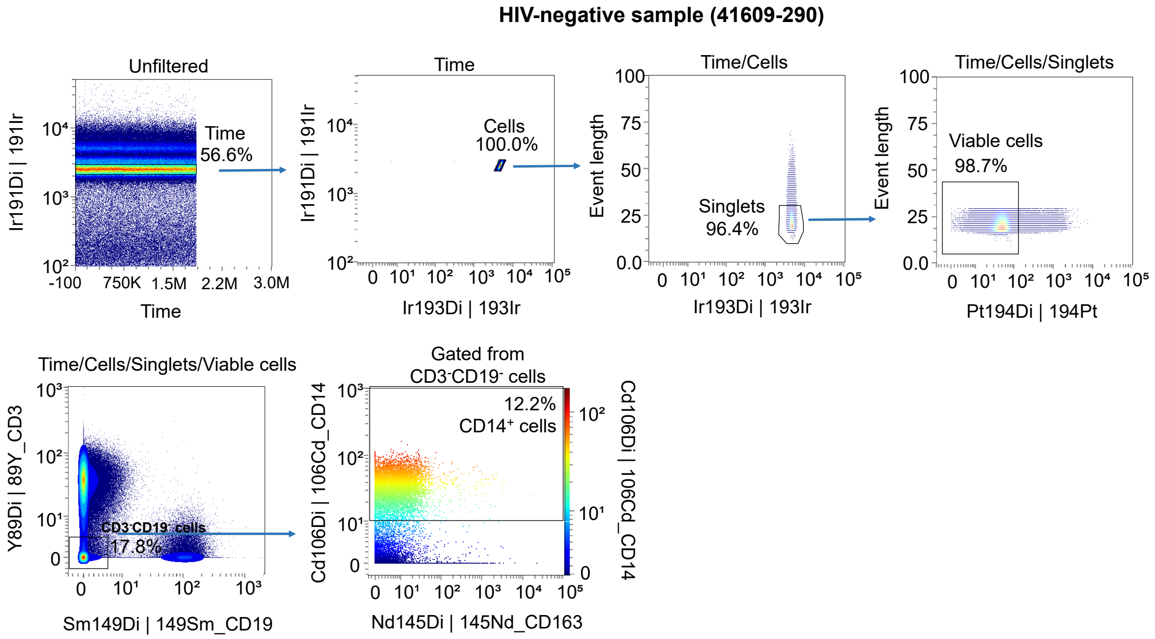

Supplementary Figure S6. Gating strategy of CD14⁺ monocytes (CD14⁺CD3⁻CD19⁻). Representative gating strategy of mass cytometry data to identify CD3⁻CD19⁻ cells from viable cells. Data shown is representative from an HIV-negative sample (41609-290). Total CD14⁺ cells were gated from live CD3⁻CD19⁻ cells. Scatter plots show selection on the CD14 marker.

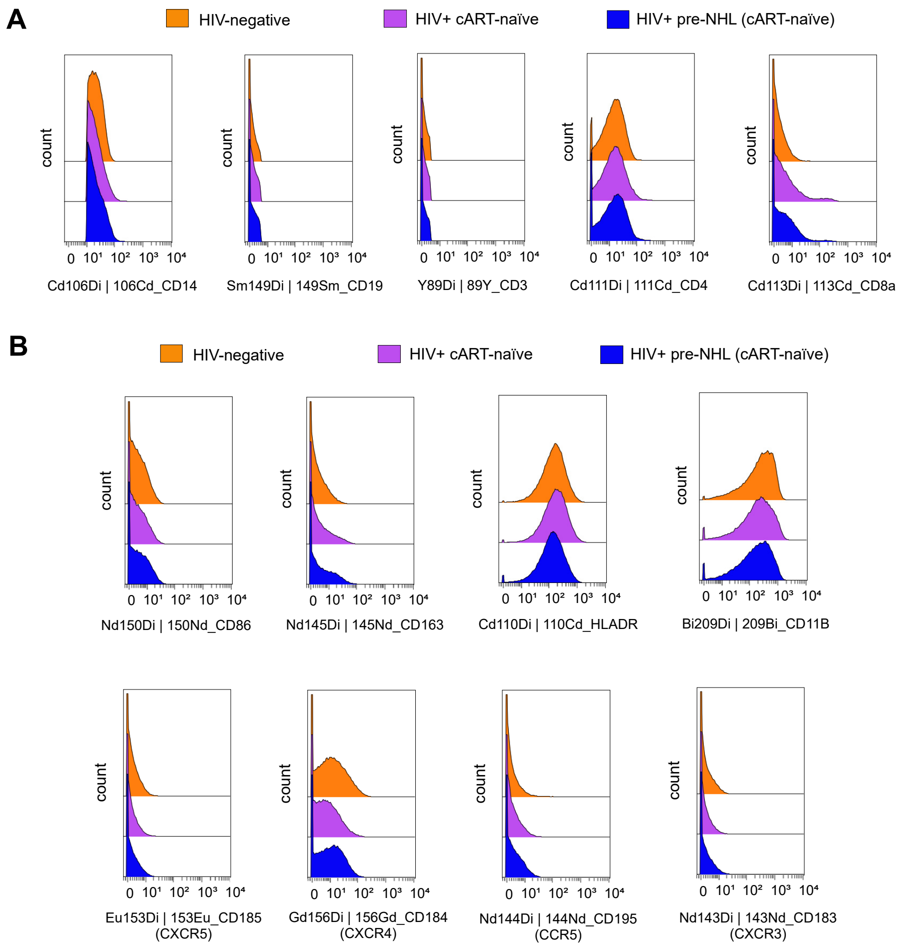

Supplementary Figure S7. Histogram plots of markers expressed on CD14^{+} monocytes (CD14^{+}CD3^{-}CD19^{-}). (**A**) Surface marker expression of CD14, CD11b, CD3, and CD19 in CD14^{+} monocytes (CD3^{-} CD19^{-}) after CyTOF of PBMCs from HIV-negative (n = 10), HIV-positive cART-naïve (n = 20), and HIV-positive pre-NHL (cART-naïve) (n = 10) cohort participants. (**B**) Surface marker expression of select markers of monocytes and macrophages (CD86, CD163, HLA-DR), CD11b, and chemokine receptors (CXCR5, CXCR4, CCR5, CXCR3).

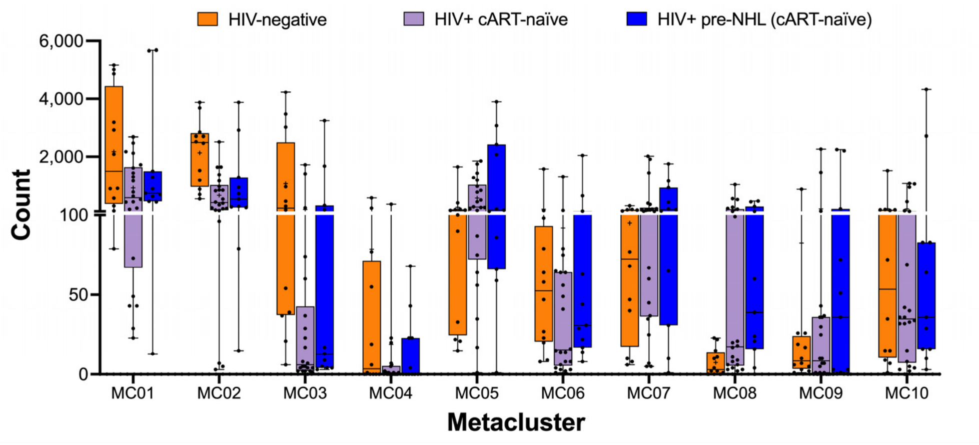

Supplementary Figure S8. Total count of CD14$^+$ monocyte (CD14$^+$CD3$^-$CD19$^-$) metaclusters. Box plots showing cell counts for each metacluster shown in **Figure 3A.**
